# Autism-linked *NLGN3* is a key regulator of gonadotropin-releasing hormone deficiency

**DOI:** 10.1242/dmm.049996

**Published:** 2023-03-28

**Authors:** Roberto Oleari, Antonella Lettieri, Stefano Manzini, Alyssa Paganoni, Valentina André, Paolo Grazioli, Marco Busnelli, Paolo Duminuco, Antonio Vitobello, Christophe Philippe, Varoona Bizaoui, Helen L. Storr, Federica Amoruso, Fani Memi, Valeria Vezzoli, Valentina Massa, Peter Scheiffele, Sasha R. Howard, Anna Cariboni

**Affiliations:** ^1^ Department of Pharmacological and Biomolecular Sciences, University of Milan, Milan 20133, Italy; ^2^ CRC Aldo Ravelli for Neurotechnology and Experimental Brain Therapeutics, Department of Health Sciences, University of Milan, Milan 20142, Italy; ^3^ Department of Health Sciences, University of Milan, Milan 20142, Italy; ^4^ Laboratory of Endocrine and Metabolic Research, IRCCS Istituto Auxologico Italiano, Cusano Milanino 20095, Italy; ^5^ Unité Fonctionnelle Innovation en Diagnostic Génomique des Maladies Rares, Fédération Hospitalo-Universitaire (FHU) TRANSLAD, CHU Dijon Bourgogne, Dijon 21079, France; ^6^ INSERM UMR 1231 GAD (Génétique des Anomalies du Développement), Université de Bourgogne, Dijon 21070, France; ^7^ Genetics and Neurodevelopment, Centre Hospitalier de l'Estran, Pontorson 50170, France; ^8^ Centre for Endocrinology William Harvey Research Institute Barts and the London School of Medicine and Dentistry, Queen Mary University of London, London EC1M 6BQ, UK; ^9^ Royal London Children's Hospital, Barts Health NHS Trust, London E1 1BB, UK; ^10^ Wellcome-MRC Cambridge Stem Cell Institute, Jeffrey Cheah Biomedical Centre, Cambridge CB2 0AW, UK; ^11^ Biozentrum of the University of Basel, Basel 4056, Switzerland

**Keywords:** GnRH neurons, Transcriptome, NLGN3, Neuritogenesis, Delayed puberty, Autism spectrum disorder

## Abstract

Gonadotropin-releasing hormone (GnRH) deficiency (GD) is a disorder characterized by absent or delayed puberty, with largely unknown genetic causes. The purpose of this study was to obtain and exploit gene expression profiles of GnRH neurons during development to unveil novel biological mechanisms and genetic determinants underlying GD. Here, we combined bioinformatic analyses of immortalized and primary embryonic GnRH neuron transcriptomes with exome sequencing from GD patients to identify candidate genes implicated in the pathogenesis of GD. Among differentially expressed and filtered transcripts, we found loss-of-function (LoF) variants of the autism-linked neuroligin 3 (*NLGN3*) gene in two unrelated patients co-presenting with GD and neurodevelopmental traits. We demonstrated that *NLGN3* is upregulated in maturing GnRH neurons and that NLGN3 wild-type, but not mutant, protein promotes neuritogenesis when overexpressed in developing GnRH cells. Our data represent proof of principle that this complementary approach can identify new candidate GD genes and demonstrate that LoF *NLGN3* variants can contribute to GD. This novel genotype–phenotype correlation implies common genetic mechanisms underlying neurodevelopmental disorders, such as GD and autistic spectrum disorder.

## Teaser

**Summary:** This study shows, for the first time, a role for *NLGN3* in GnRH neuritogenesis and reveals a common genetic mechanism between GnRH deficiency and autism, by applying a combined multiomic approach.

## INTRODUCTION

Gonadotropin-releasing hormone (GnRH, encoded by *GNRH1*) is the master hormone regulating the hypothalamic–pituitary–gonadal (HPG) reproductive axis, and its pulsatile secretion is crucial for puberty onset, sexual maturation and fertility (Herbison, 2016). GnRH is produced by a small number of hypothalamic neuroendocrine neurons called GnRH neurons, which, during embryonic development, originate in the nasal placode and migrate along vomeronasal nerves to reach the hypothalamus (Oleari et al., 2019).

Disruption in GnRH neuron development or hypothalamic function are the leading causes of genetic reproductive disorders such as hypogonadotropic hypogonadism (HH) and Kallmann syndrome, which are characterized by absent or delayed puberty, owing to GnRH deficiency (GD) (Boehm et al., 2015). The known genes causing GD account for only 50% of cases (Boehm et al., 2015; Oleari et al., 2021), supporting the need for studying the genetic signatures of GnRH neurons as a novel strategy to expedite etiological discovery in the remaining cases. However, the study of the GnRH neuronal system is hampered by the difficulty in obtaining primary GnRH neurons, which are small in number and lack specific markers. To overcome these issues, alternative tools, such as immortalized GnRH neuron cell lines (Maggi et al., 2000) and reporter rodent and zebrafish lines (Messina et al., 2016; Kato et al., 2003; Abraham et al., 2009), have been adopted.

Recently, RNA sequencing has been applied to obtain the transcriptome of induced pluripotent stem cell (iPSC)-derived human GnRH neuron progenitors and early postmitotic GnRH neurons (Lund et al., 2020; Keen et al., 2021; Wang et al., 2022). Yet, no reports of gene expression profiles of immortalized or primary GnRH neurons during the entire developmental process are available.

Here, we obtained, for the first time, the transcriptomic profiles of rodent immortalized and primary embryonic GnRH neurons. Further, by combining filtering strategies with exome sequencing from human patients and *in vitro* functional experiments, we identified neuroligin 3 (*NLGN3*) as a new GD-disease candidate gene. *NLGN3* belongs to the neuroligin family, a class of postsynaptic cell adhesion molecules that regulate synapse organization (Uchigashima et al., 2021) and dendritic outgrowth (Xu et al., 2019), and *NLGN3* missense variants have previously been associated with autistic spectrum disorder (ASD) (Nguyen et al., 2020). In this work, we have described two patients carrying novel nonsense *NLGN3* variants and presenting with clinical phenotypes of GD and ASD, therefore providing the first genetic correlation between these two neurodevelopmental disorders, which was previously only implied by a registry-based association study (Oleari et al., 2021). Moreover, we have demonstrated that NLGN3 is important for GnRH neuronal development, with deficiency resulting in defective neuritogenesis in a cellular model.

## RESULTS

### Analysis of mouse immortalized GnRH neuron transcriptome reveals stage-specific expression signatures

**Fig. 1.**
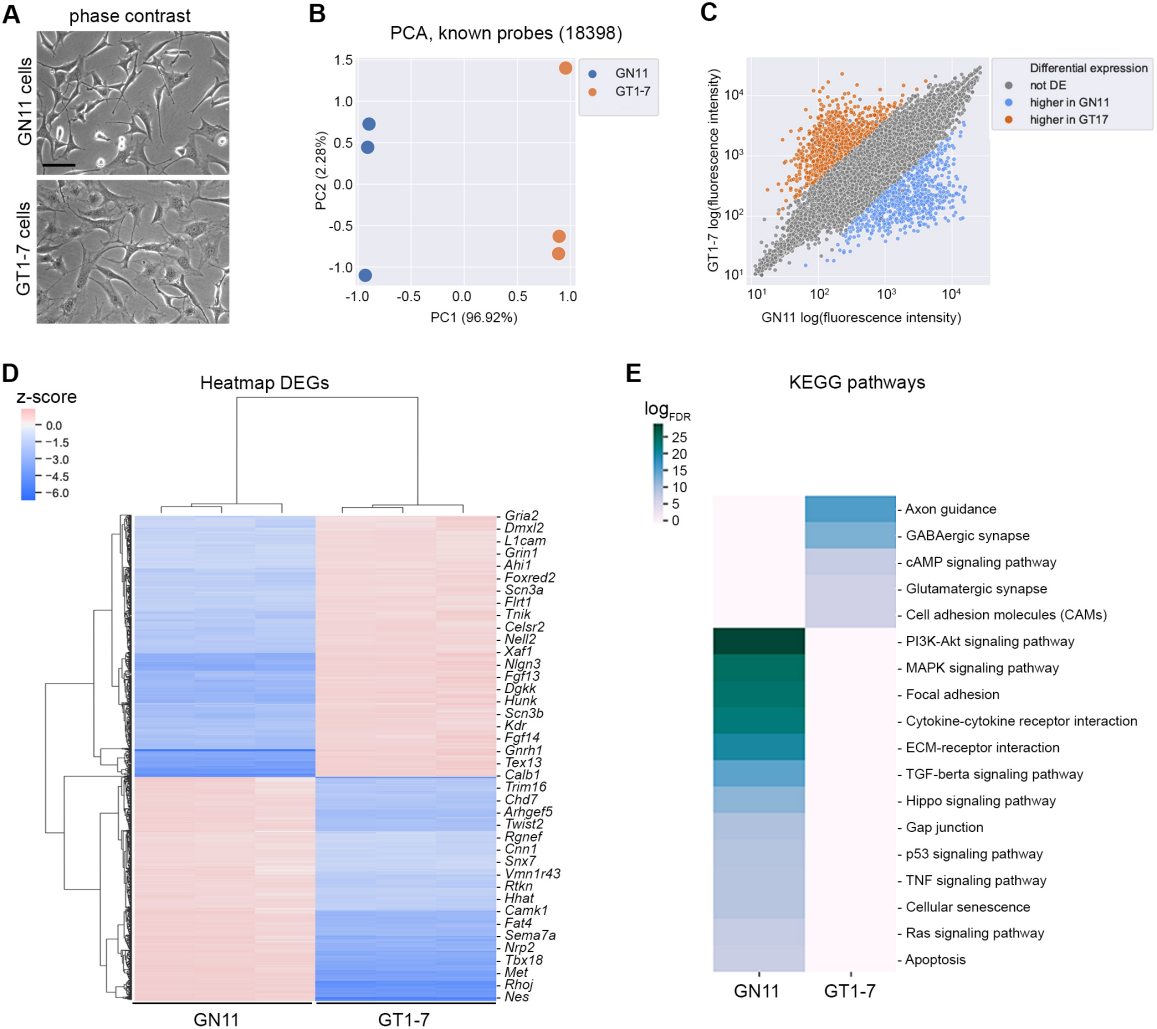
**Transcriptomic analysis of GN11 and GT1-7 immortalized gonadotropin-releasing hormone (GnRH) neurons.** (A) Phase-contrast microphotographs of GN11 and GT1-7 cells. Scale bar: 125[Supplementary-material sup1]μm. (B) Dimensionality reduction was performed on gene expression space (annotated probes only) for each GN11 and GT1-7 cell sample, and the first two principal coordinates (PCs) are charted. PCA, principal component analysis. (C) Scatter plot showing differentially expressed genes (DEGs) (log_FC_>2, adjusted *P*-value<0.05) between GN11 and GT1-7 cells. GN11 upregulated genes are indicated in blue, whereas GT1-7 upregulated genes are in orange; gray dots indicate non-DEGs. (D) The ratio of the log_2_ expression value and mean value across all samples for each probe has been calculated and clustered hierarchically (Euclidean distance metric) for DEGs (log_FC_>2) between GN11 and GT1-7 cells. Red indicates higher expression; blue indicates lower expression. (E) Enriched Kyoto Encyclopedia of Genes and Genomes (KEGG) pathways [false discovery rate (FDR)<0.01] found by STRING functional enrichment analysis computed on DEGs between GN11 and GT1-7 cells. Enrichment scores are reported as log_FDR_; higher values (deep blue) indicate highly enriched pathways, lower values (gray) indicate poorly enriched pathways.

We first obtained the transcriptomic profiles of GN11 and GT1-7 cells, which represent well-characterized immortalized GnRH neuron cell lines, by using an Affymetrix GeneChip array (*n*=3; GSE174902; Fig. 1A). These neurons, which derive from mouse tumors localized in the olfactory bulb and in the hypothalamus, respectively, are characterized by different cellular properties, namely migration for GN11 cells and GnRH hormone secretion for post-migratory GT1-7 cells (Maggi et al., 2000). Sample clustering by principal component analysis (PCA) revealed a high degree of similarity within samples of each cell line (Fig. 1B). Among the two cell lines, 1612 differentially expressed probes (|log_FC_|>2, adjusted *P*<0.05; Fig. 1C,D), mapping to 1515 unique annotated genes, were found. Specifically, 703 genes were significantly upregulated in GN11 cells, while GT1-7 cells showed a significant overexpression of 812 genes. Functional enrichment analysis, performed with reString software (Manzini et al., 2021) and based on Kyoto Encyclopedia of Genes and Genomes (KEGG) pathway annotations, revealed significantly enriched pathways [false discovery rate (FDR)<0.01] that were consistent with the different biological properties of the two cell lines (Fig. 1E). In particular, genes upregulated in GN11 cells significantly populated pathways related to migration (Fig. S1A). Conversely, the most represented pathways in GT1-7 cells belonged to neuronal maturation processes (e.g. axon development and synapse assembly) (Fig. S1B). Similar results were obtained with the functional enrichment analysis of Gene Ontology (GO) biological processes (BP) (FDR<10^−5^; Fig. S1C). Overall, these analyses identified that a high number of maturational stage-specific genes enriched biological pathways relevant to and consistent with the different phases of GnRH neuron development, such as migration at early phases (GN11) and establishment of neuron projections and synapse development at later phases (GT-7) (Forni and Wray, 2015; Cho et al., 2019).

### Combined data mining of immortalized and primary GnRH neuron transcriptomes with GD-causative genes prioritizes 29 candidate genes

**Fig. 2.**
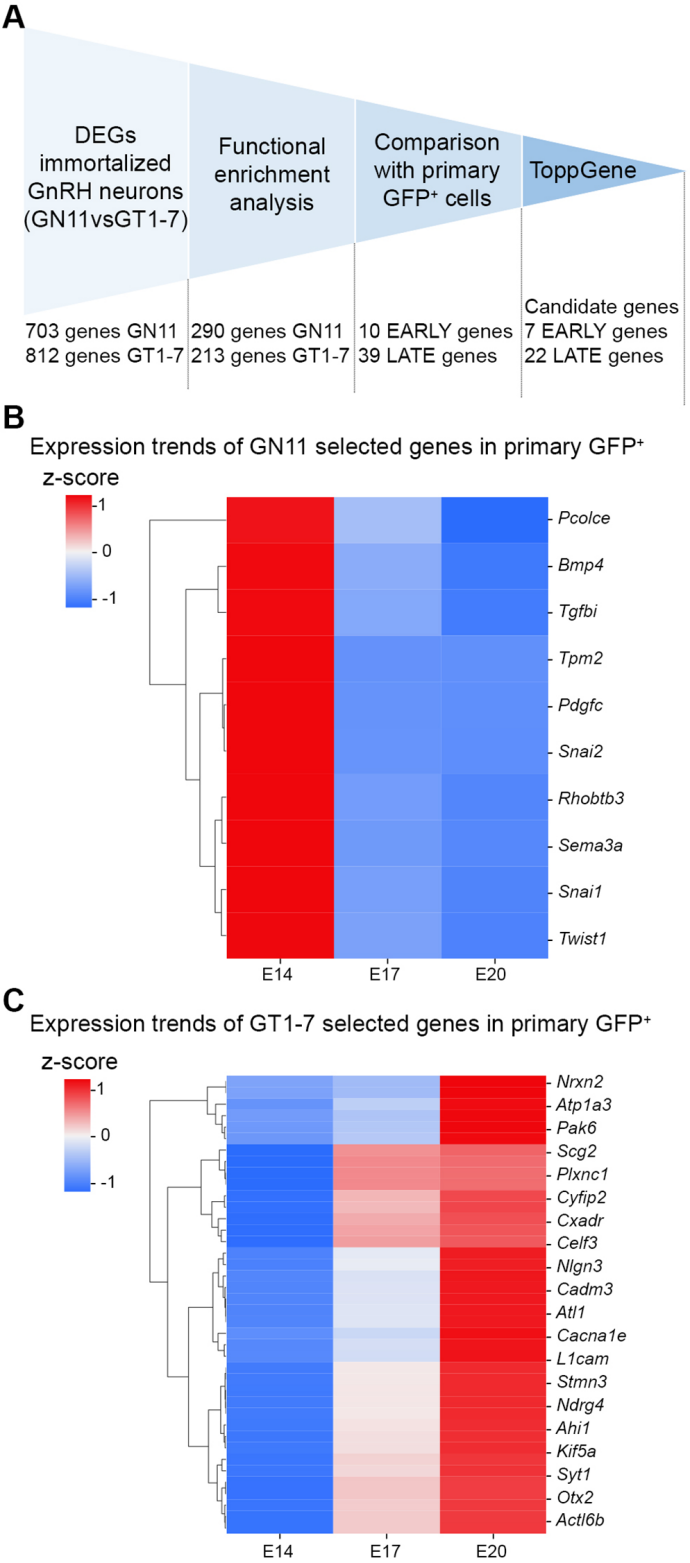
**Filtering analyses revealed 29 candidate genes that differently enriched early and late developmental stages.** (A) Flowchart of filtering strategy applied to narrow down the list of candidate genes. Upregulated genes in GN11 and GT1-7 cells were filtered upon selection of Gene Ontology (GO)/KEGG pathways related to cytoskeleton dynamics, migration and axon development (see Tables S1 and S2 for the full list of pathways). The expression trends of selected genes were validated in primary GFP^+^ neurons; only genes that display decreasing or increasing expression with development were selected. Last, candidate genes were prioritized with ToppGene software, and genes with overall *P*<0.01 from both groups were used for subsequent analyses. (B,C) *Z*-scored gene expression values are shown for genes belonging to selected KEGG pathways that exhibited decreasing (B; early genes) or increasing (C; late genes) expression trends with embryonic stage in primary GFP^+^ cells. Heatmaps represent color-coded gene expression levels.

To refine the list of candidate genes, we leveraged an integrated approach based on functional enrichment analysis, transcriptomic profiles of primary GnRH neurons and prioritization bioinformatic tools (Fig. 2A).

We first selected genes belonging to significantly enriched pathways (FDR<0.01 for KEGG pathways and FDR<10^−5^ for GO BP), which related to cytoskeleton dynamics, migration and axon development for each cell line (Tables S1 and S2).

Then, to confirm the biological relevance of selected genes *in vivo*, we integrated these gene lists with those obtained by transcriptomic profiling of primary GnRH neurons isolated from fluorescence-activated cell sorting (FACS)-sorted *Gnrh1*-GFP rat embryos (Kato et al., 2003) at embryonic day (E)14, E17 and E20 (*n*=1 per each stage; GSE174896; Fig. S2A,B). Owing to technical constraints, RNA from primary neurons from different embryos was pooled and run as a single replicate, for each stage. By examining GFP^+^ cell transcriptomes using the E14 stage as a common baseline, we identified expression changes in 4844 detected probes, mapping to 2739 unique annotated genes (|log_FC_|>1; Fig. S2C,D), including genes previously implicated in GnRH neuron development by direct experimental evidence (e.g. *Sirt1*, *Reln*, *Cxcr7*, *Slit2*, *Lif*) (Cho et al., 2019; Cariboni et al., 2007) or found mutated in GD patients (e.g. *SEMA3A*, *SEMA7A*, *FGFR1*, *NRP1*) (Boehm et al., 2015). Interestingly, each developmental stage was characterized by a unique transcriptomic signature (Fig. S3) that exhibited a high level of overlap with the ones observed in immortalized neurons (Fig. S4). The two datasets were then integrated to obtain two gene set clusters, containing genes from selected enriched pathways of GN11 and GT1-7 cells that exhibited a decreasing (‘early’ genes) or increasing (‘late’ genes) expression trend along with embryonic age in primary GFP^+^ cells (Fig. 2B,C).

Last, to identify, among filtered genes, those that could be potentially implicated in GD pathogenesis, ToppGene software (Chen et al., 2009) was applied to rank candidates based on functional or structural similarity with a set of known GD causative ‘input’ genes (Boehm et al., 2015; Maione et al., 2018) (Table S3). The top output genes for each list (early or late) with an overall *P*-value less than 0.01 were selected, thus obtaining seven early and 22 late genes (Table 1). The expression levels of two representative early and late genes were validated in GN11 and GT1-7 cells by reverse transcription quantitative PCR (RT-qPCR) (Fig. S5).

**Table 1.**
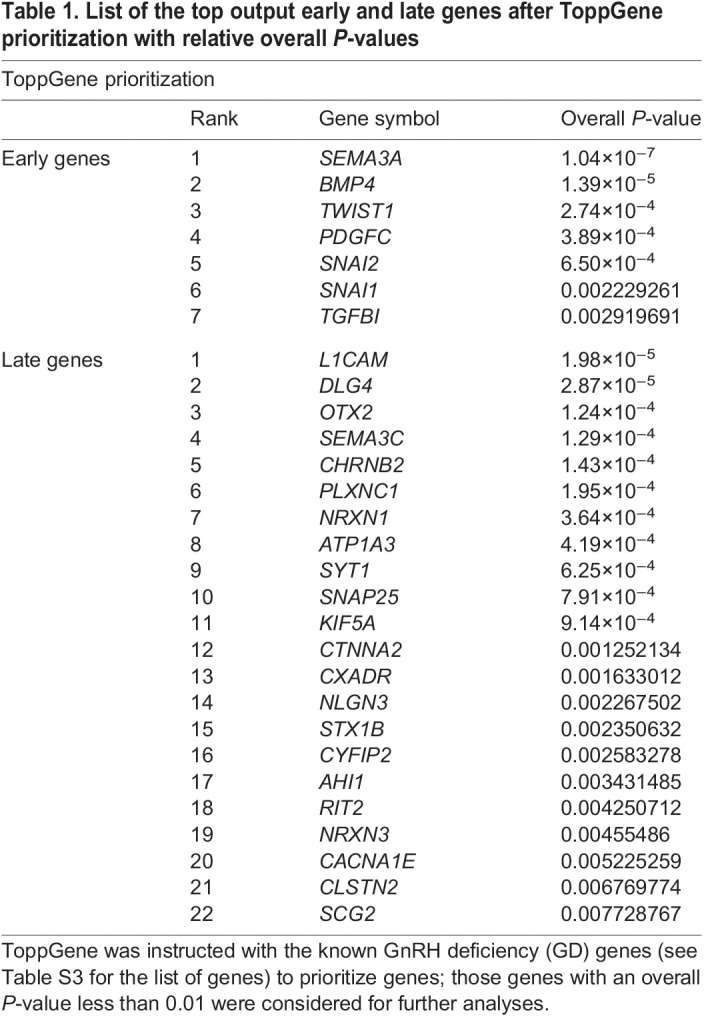
List of the top output early and late genes after ToppGene prioritization with relative overall *P*-values

In summary, the microarray analyses combined with candidate gene-filtering strategies facilitated the identification of 29 candidate genes that, with the exception of *SEMA3A*, have not been previously linked with GnRH neuron biology or GD.

### *NLGN3* candidate gene is mutated in two patients affected by GD and ASD

**Fig. 3.**
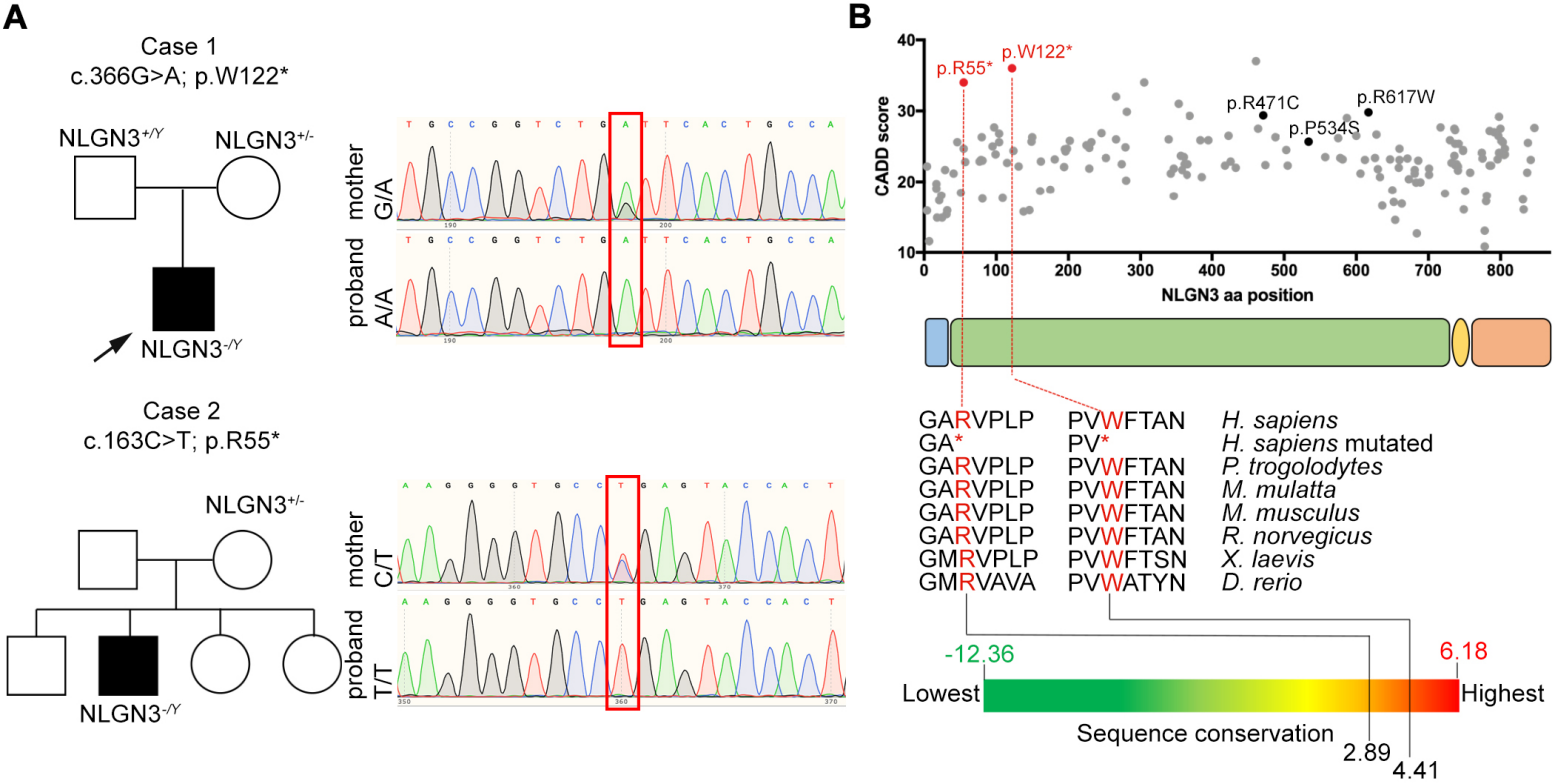
**Two novel *NLGN3* nonsense variants found in patients with GnRH deficiency (GD) and autism spectrum disorder (ASD).** (A) Family pedigrees of the two probands (Case 1 and Case 2) identified in this study and presenting GD and ASD features. Sequence chromatograms of nucleotides 70367955-70367975 and nucleotides 70367752-70367772 of the *NLGN3* coding sequence in Case 1, Case 2 and their unaffected mothers (the positions of the C>T and G>A are highlighted by the red boxes). (B) Schematic representation of NLGN3 protein (encoded by the NM_181303.1 transcript): signal peptide (light blue), extracellular (green), transmembrane (yellow) and intracellular (orange) domains. All missense variants (gray dots) reported in gnomAD database (v2.1.1) are plotted according to their amino acid (aa) position and Combined Annotation-Dependent Depletion (CADD) score. Pathogenic variants related to ASD are indicated in black: R471C (Jamain et al., 2003), P534S (Quartier et al., 2019) and R617W (Redin et al., 2014). Identified variants (R55* and W122*) are indicated in red and have a higher CADD score compared to others. Multi-species alignment of partial protein sequences of vertebrate NLGN3 ortholog proteins shows that the R55 and W122 residues are evolutionarily conserved in humans and other vertebrate species with a high conservation degree, calculated by GERP++.

To validate the relevance of the top 29 genes in human GD pathogenesis, the presence of potentially deleterious variants for each gene was interrogated in exomes from our cohort of GD patients (*n*=47) (Saengkaew et al., 2021). Only variants that met the American College of Medical Genetics (ACMG) criteria for pathogenicity, likely pathogenicity or variants of uncertain significance were retained in the analysis. Overall, we found three variants with Combined Annotation-Dependent Depletion (CADD) >25 and minor allele frequency <1% (Table S4). Of these, only one predicted loss-of-function (LoF) variant was identified, in the X-linked gene *NLGN3* (Gene ID 5441). A male patient (Case 1) with partial GD [with puberty that had initiated and then arrested (Ferreira et al., 2013)] and ASD traits was found to carry a stop-gain variant in *NLGN3* (Fig. 3A). By interrogating GeneMatcher (Sobreira et al., 2015), we found an additional *NLGN3* LoF variant in an unrelated patient with phenotypic features of GD and ASD with intellectual disability (Case 2; Fig. 3A).

Case 1 was found to be hemizygous for a novel stop-gain variant in *NLGN3* (NM_181303.1: c.366G>A, p.W122*; VCV002443311.1) (Fig. 3A,B). He was born to healthy non-consanguineous parents, and his mother was found to be heterozygous for the same variant, as confirmed by Sanger sequencing (Fig. 3A). Clinically, the proband presented in early adulthood with a picture of pubertal arrest with biochemical evidence of GD (Table 2). The proband reported late onset of puberty and had achieved testes volume of 10 ml by the age of first review. He was commenced on treatment with testosterone esters, which were gradually increased up to a maximal dose of 250 mg every 4 weeks, but testes volume had increased only to 12 ml at the age of more than 20 years, consistent with partial GD phenotype (Boehm et al., 2015). In addition, he had associated phenotypic features including obesity [body mass index (BMI), 36; height, 178.5 cm], depression, anxiety, and social and communication difficulties consistent with ASD. Inhibin B concentrations were in the low-normal range. Mildly elevated thyroid-stimulating hormone (TSH) concentrations with low-normal free thyroxine (T4) were reported, likely secondary to obesity (Table 2). Magnetic resonance imaging scan showed a normal appearance of the pituitary gland, pituitary stalk and hypothalamus (Fig. S6). There was no family history of GD, but his mother, who carried the same variant in *NLGN3*, was also obese.

**Table 2.**
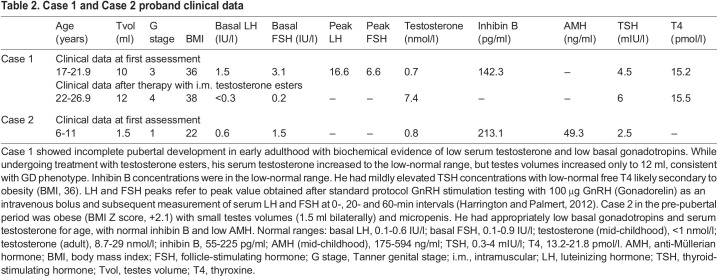
Case 1 and Case 2 proband clinical data

Case 2 (NM_181303.1: c.163C>T; p.R55*; VCV002442803.1) is a pre-pubertal boy who presented with stereotypia, learning difficulties and ASD features. In addition, he was noted to have micropenis (untreated penile length 2.5 cm in mid-childhood) and absent erections, which are known predictive signs of HH in pediatric males (Boehm et al., 2015; Swee and Quinton, 2019). At the age of 7-12 years, he was obese (BMI Z-score, +2.1; height, 148 cm) with small testes volumes (1.5 ml bilaterally). His mother also had history of learning difficulties and stereotypic behaviors, but there was no family history of GD. He had appropriately low gonadotropins and testosterone for age when first tested in mid-childhood, with normal inhibin B and low anti-Müllerian hormone (AMH) (Table 2).

In both cases, exome sequencing of the probands excluded deleterious variants in known GD genes, and no other variants of interest were identified in relevant genes, e.g. related to developmental delay. Sanger sequencing of the probands' mothers confirmed them to be the heterozygous carrier in each family, consistent with an X-linked recessive inheritance pattern. DNA from other Case 2 family members was not available.

Interestingly, both variants cause premature stop codons within the *NLGN3* extracellular domain, resulting in truncated proteins that are likely to be dysfunctional. Both variants are novel, not found in the gnomAD database (v2.1.1, accessed 6 February 2022), and, notably, the *NLGN3* gene is highly intolerant to protein-truncating changes (pLi, 0.98). According to the ACMG (Richards et al., 2015), these variants are both classified as pathogenic (Table 3). Bioinformatic tools predicted these variants to be damaging, deleterious and disease causing, with CADD score >35 and DANN score >0.99 (Fig. 3B and Table 3). Further, multi-species protein alignment combined with GERP++ software showed that identified variants affect highly conserved residues (Fig. 3B).

**Table 3.**
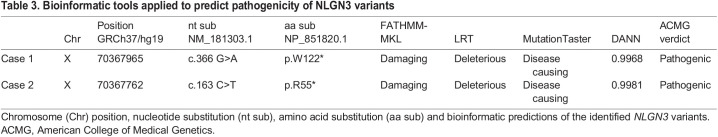
Bioinformatic tools applied to predict pathogenicity of NLGN3 variants

### *NLGN3* is upregulated in maturing GnRH neurons, and *NLGN3* truncating variants impair protein synthesis and neurite outgrowth *in vitro*

**Fig. 4.**
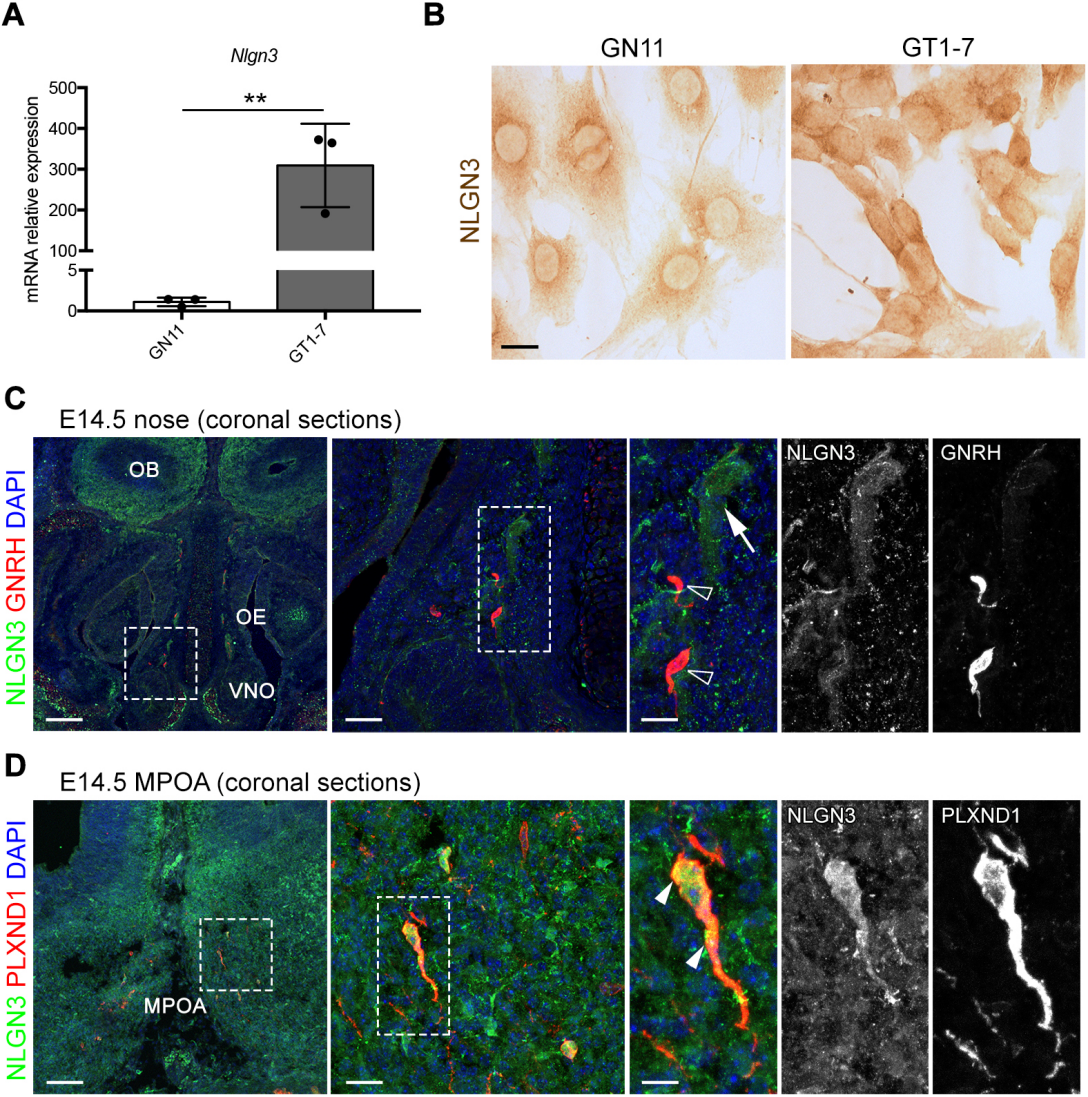
**NLGN3 is developmentally regulated in GnRH neurons.** (A) Quantitative PCR analysis performed on GN11 and GT1-7 cells revealed higher *Nlgn3* expression levels in GT1-7 cells (log_FC_=8.12, *P*<0.01). This result is in line with microarray experiments (log_FC_=4.15; *P*<0.00001). Data are presented as mean±s.d. of three biological replicates. Unpaired two-tailed Student's *t*-test (**P<0.01). (B) Immunoperoxidase staining for NLGN3 on GN11 and GT1-7 revealed different levels of endogenous NLGN3 protein in these cells. Scale bar: 25 μm. (C,D) Coronal sections of E14.5 mouse heads were immunolabeled for NLGN3 together with GnRH (C) or PLXND1 (D) to detect GnRH neurons. Sections are shown at the level of the VNO (nose; C) or MPOA (forebrain; D). White dashed line boxes indicate areas shown at higher magnification on the right of the corresponding panel, with single channels also shown adjacent to the panel. Open arrowheads indicate examples of GnRH-positive cells that lack NLGN3; filled arrowheads indicate examples of GnRH-positive cells with NLGN3. Arrows indicate examples of NLGN3-positive cells in the nasal parenchyma. Sections were counterstained with DAPI. MPOA, medial preoptic area; OB, olfactory bulb; OE, olfactory epithelium; VNO, vomeronasal organ. Scale bars: 250 μm (right panels), 150 μm (middle panels) or 50 μm (left panels).

Our transcriptomic analyses revealed that the X-linked *NLGN3* gene was upregulated in primary and immortalized GnRH neurons during development. Thus, to validate its developmentally regulated expression, we performed the following experiments. First, we confirmed higher *NLGN3* levels in maturing GT1-7 versus immature GN11 cells (Fig. 4A,B), by RT-qPCR and immunocytochemistry, using a previously validated anti-NLGN3 antibody. Then, we analyzed NLGN3 expression in E14.5 mouse sections and confirmed colocalization of NLGN3 protein in GnRH neurons that had settled in the maturing hypothalamus (Fig. 4C).

**Fig. 5.**
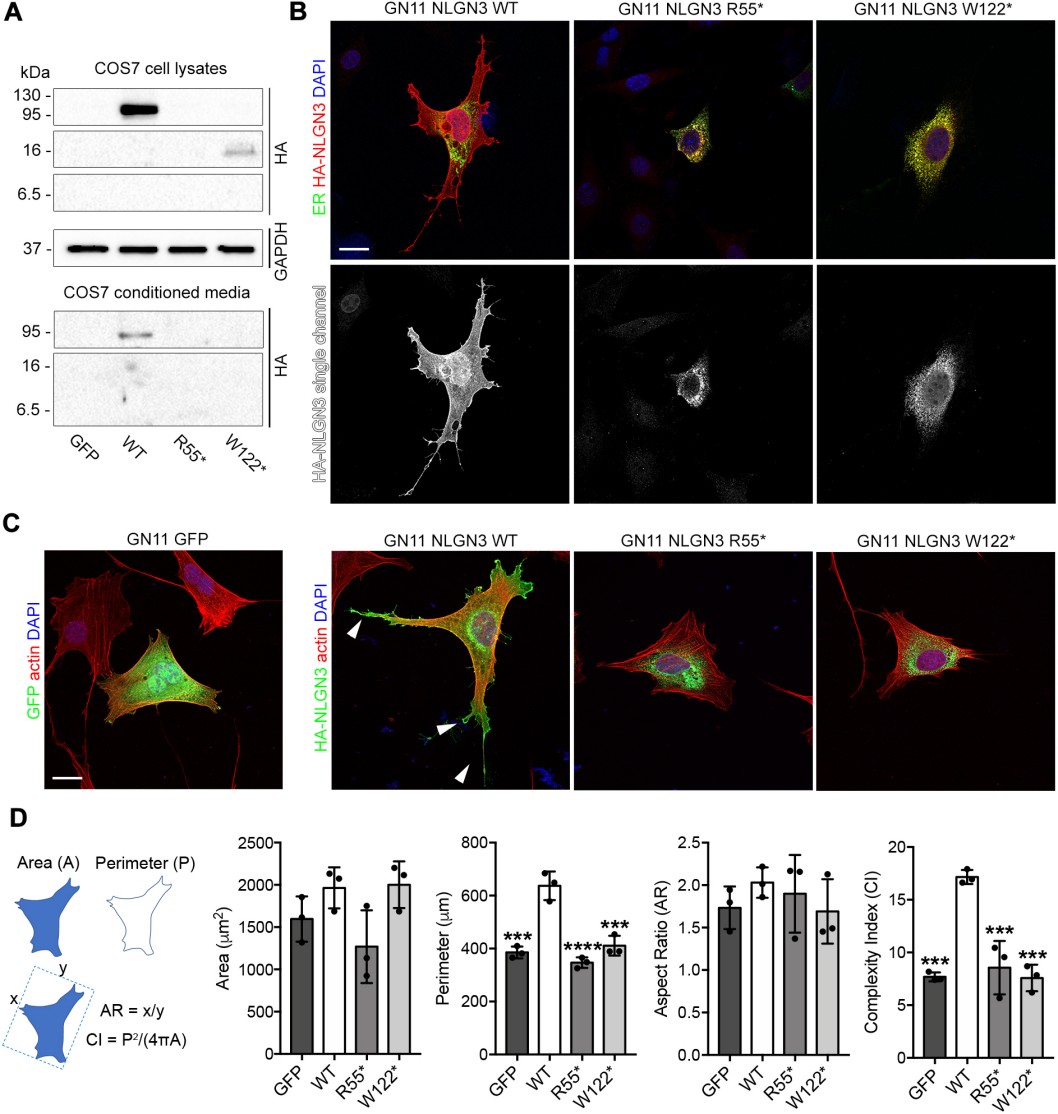
**Mutant NLGN3 proteins induce endoplasmic reticulum retention and impair neuritogenesis in immortalized GnRH neurons.** (A) Immunoblot analysis with anti-HA or anti-GAPDH antibodies on whole-cell lysates and conditioned media from COS7 cells overexpressing control vector (GFP), HA-tagged wild-type (WT) or mutant NLGN3. GAPDH is shown as a loading control for cell lysates. (B) Confocal images of GN11 cells transfected cells with mEmerald-ER-3 (green) and human WT or mutated HA-tagged NLGN3 vectors and stained for HA (red) after 24 h. HA-NLGN3 single-channel images are shown below each panel. (C) Confocal images of GN11 transfected with human WT or mutated NLGN3 HA-tagged encoding vector and stained for HA (green) and F-actin (red). Arrowheads point at neurites in NLGN3 WT-expressing GN11 cells. (D) Quantification of cell perimeter (P), cell area (A), aspect ratio (AR) and complexity index (CI) in GN11 cells transfected with indicated plasmids. Column graph quantifications show a significant increase in the number of neurites extending from NLGN3 WT-expressing cells compared to NLGN3 mutants and GFP-transfected control cells. Data are presented as mean±s.d. of three biological replicates. Significant differences were determined by one-way ANOVA followed by Tukey's multiple comparison test (***P<0.001; ****P<0.0001). Nuclei were counterstained with DAPI. Scale bars: 25 μm.

To functionally validate the predicted pathogenicity of the identified *NLGN3* variants, we overexpressed human hemagglutinin (HA)-tagged wild type (WT) (Chih et al., 2004), R55* or W122* NLGN3 in cell culture models. Initially, protein synthesis and secretion of WT versus mutated NLGN3 proteins were evaluated in COS7 cells, by immunoblotting analysis on lysates and conditioned media, respectively. As expected, a 110 kDa band corresponding to the full-length protein was observed in lysates of COS7 cells transfected with NLGN3 WT (Fig. 5A) (Bemben et al., 2019). In contrast, R55* and W122* variants led to the formation of prematurely truncated proteins of expected molecular masses (7 and 14 kDa, respectively) (Fig. 5A). In addition, a shorter form (90 kDa) of NLGN3 WT, which likely represents the ectodomain released by proteolytic shedding (Bemben et al., 2019), was detected in conditioned media. We could not detect equivalent shed or secreted forms for the NLGN3 mutant proteins (Fig. 5A). To study localization of these mutant proteins, we performed similar overexpression experiments and immunocytochemical analysis in GN11 cells, which show low levels of endogenous NLGN3 (Fig. 4A,B). Immunofluorescence staining for HA in GN11 cells revealed that mutant proteins were only detected in the endoplasmic reticulum (ER), selectively labeled with mEmerald-ER-3 vector (Fig. 5B). By comparison, the WT protein was efficiently transported to the plasma membrane. Of note, we observed only very few HA^+^ cells when transfected with R55* NLGN3, strongly supporting rapid NLGN3 protein degradation and consequent lack of detection, as previously described for other NLGN3 mutant proteins (Quartier et al., 2019). Altogether, these observations strongly support a LoF effect of the two newly identified variants.

Because NLGN3 promotes neuritogenesis in human neural progenitor cells (Gatford et al., 2022), we studied the effect of WT or mutant NLGN3 proteins on GnRH neuron morphology and neurite outgrowth by overexpression experiments in GN11 cells, as previously described (Bouilly et al., 2018). NLGN3 WT promoted the formation of several protrusions, with cells displaying an increased spreading compared to GFP-transfected controls (Fig. 5C). By contrast, the two mutants (R55* and W122*) were unable to induce such changes (Fig. 5C), with transfected cells displaying a significantly lower cell perimeter, although total area and cell polarity (i.e. aspect ratio) were not affected (Fig. 5D). Further, cells overexpressing NLGN3 mutants exhibited a significantly reduced cell shape complexity (i.e. complexity index) compared to NLGN3 WT, revealing a strong defect in cell protrusion formation (Fig. 5D). Together, these results confirmed that the identified NLGN3 variants are LoF and failed to induce cytoskeletal and membrane rearrangements typical of neuritogenesis.

## DISCUSSION

In this work, we have interrogated the transcriptomic profiles of rodent immortalized and developing primary GnRH neurons and developed a new resource for understanding the key developmental regulation of GnRH neurons. Further, we have identified NLGN3 as modulator of GnRH neuron neuritogenesis and novel *NLGN3* LoF variants in two unrelated patients with GD and ASD traits.

Previous works have generated transcriptomes from human iPSC- or embryonic stem cell-derived GnRH-like cells by RNA sequencing (Lund et al., 2020; Keen et al., 2021) and, very recently, at single-cell level (Wang et al., 2022). Although caution should be exercised in transcriptomic comparative analyses between different models and sequencing techniques, our transcriptomic data highlighted the presence of consistent expression trends in the majority of our 29 candidate genes across GnRH neuron clusters recently identified by a single-cell RNA-sequencing approach in human iPSC-derived GnRH neurons (Wang et al., 2022). Further, candidate genes generated from our analyses have been experimentally associated with GnRH neuron development (e.g. *Bmp4*, *Otx2*, *Plxnc1*) (Forni et al., 2013; Parkash et al., 2015; Diaczok et al., 2011) or with GD pathogenesis (e.g. *SEMA3A*) (Oleari et al., 2021), confirming the ‘bona-fide’ nature of immortalized and primary GnRH neurons as complementary models for studying physiological and pathological mechanisms of GnRH neuron development.

As an exemplar, our integrated omic analysis allowed us to identify *NLGN3* as a new candidate GD gene. Specifically, the identified variant in our Case 1 proband induces a premature stop codon in *NLGN3*, a gene that has been linked so far to ASD (Nguyen et al., 2020; Quartier et al., 2019; Jamain et al., 2003; Redin et al., 2014) but not to GD. Notably, the second patient identified in this study (Case 2) is also hemizygous for a nonsense *NLGN3* variant and, although prepubertal, displayed typical red flag signs of hypogonadism, including small testes volume and micropenis (Boehm et al., 2015; Swee and Quinton, 2019). In addition, these two probands had behavioral difficulties including ASD features or neurodevelopmental disorder (NDD), supporting the existence of a shared pathophysiological link between GD and other developmental disorders, as hypothesized by a recent phenotypic population study showing an increased risk of NDDs in patients with GD (Ohlsson Gotby et al., 2019). This implies that NLGN3 is essential for the development or function of GnRH neurons, besides its well-known role in brain neurotransmission, which is thought to be dysfunctional in ASD (Nguyen et al., 2020). Although its expression has been reported in the developing telencephalon of chick and zebrafish embryos in territories relevant to GnRH neuron development (Davey et al., 2010; Paraoanu et al., 2006), the role of NLGN3 in this system is not yet known.

In this context, because NLGN3 is developmentally upregulated both in immortalized and primary GnRH neurons and its overexpression in GN11 cells promotes neuritogenesis, NLGN3 may be required to promote neurite extension during the final phases of GnRH neuron development *in vivo*. Consequently, NLGN3 loss might have an impact on this process, thus causing GD. Interestingly, compound double *Nlgn1/3-* or *Nlgn2/3*-null mice display a reduced reproductive rate (Varoqueaux et al., 2006), in addition to behavioral phenotypes related to ASD (Varghese et al., 2017), strongly supporting a role for NLGN3 in the reproductive axis. Yet, future detailed phenotypic analyses of GnRH neuron development in NLGNs single and compound null mice will be necessary to define the exact function of NLGN3 in these systems.

Our study also links, for the first time, nonsense *NLGN3* variants to a combined phenotype of ASD and GD. Most ASD patients with *NLGN3* mutations carry missense variants, with only two previous reports of nonsense variants without functional characterization or phenotypic correlation (Nguyen et al., 2020). Further, although we cannot exclude that the identified nonsense *NLGN3* variants undergo degradation *in vivo* due to the nonsense-mediated decay mechanism of RNA surveillance, our data still provide evidence that if this mechanism does not occur, remaining NLGN3 mutant proteins are non-functional, and their expression leads to a relevant cellular phenotype.

Although the association of truncating *NLGN3* variants with the co-existence of GD with ASD or NDD is being supported so far only by the two cases reported in our study, it will be interesting to expand genotype–phenotype correlation to other cases and to understand the molecular mechanisms underlying this association. Interestingly, growing evidence supports the existence of common genetic determinants between NDDs and GD, as shown by a 2022 study linking *SOX11* mutations to a novel NDD with HH (Al-Jawahiri et al., 2022). It is known that GnRH neurons also send projections to extrahypothalamic areas, including brain regions involved in intellectual functions (Casoni et al., 2016). A very recent study showed that GnRH treatment is effective in restoring cognitive performance in patients with Down syndrome (Manfredi-Lozano et al., 2022). Thus, we speculate that NLGN3 might also have a role in GnRH neuron neuritogenesis in non-hypothalamic regions, thus contributing to the neurodevelopmental phenotype directly via GnRH neurons as well as via non-GnRH networks.

Overall, our study, by exploiting the power of omic analyses, has provided a novel experimental resource that will be valuable for the identification of the elusive mechanisms underlying GnRH neuron biology and related diseases. Further, our findings provide the first proof of principle that the transcriptomic analysis of immortalized and primary GnRH neurons, combined with human genetics, *in silico* tools and *in vitro* models, may be applied to identify novel GD candidate genes. In the future, mutational screening of our top 29 genes in other patient cohorts could identify novel genetic determinants of GD; of note, *PLXNC1* and *CLSTN2* variants of interest in single patients from our cohort were found. Lastly, because our studies imply the existence of a shared genetic pathway between ASD and GD, it will be important for clinicians to be aware of the potential for pubertal and reproductive disorders in children with ASD/NDD to identify those who might require hormonal therapy intervention and, vice versa, to assess the psychological and social aspects of GD-affected children reporting ASD or developmental delay.

## MATERIALS AND METHODS

### Animals

*Gnrh1*-GFP rat embryos (Kato et al., 2003) were used in Wistar background to isolate GnRH-GFP neurons. WT C57/Bl6 mouse embryos were used for expression studies (Italian Ministry of Health, license 5247B.N.QPE). To obtain embryos of defined gestational stages, rats and mice were mated in the evening, and the morning of vaginal plug formation was counted as E0.5.

### Cell lines

GN11, GT1-7 cells (Maggi et al., 2000) and COS7 cells were grown as a monolayer at 37°C in a humidified CO_2_ incubator in complete Dulbecco's modified Eagle medium (Euroclone) supplemented with 10% fetal bovine serum (FBS; Invitrogen). Subconfluent cells were harvested by trypsinization and cultured in 57 cm^2^ dishes. Cells within six passages were used for all experiments.

### Cell dissociation and FACS analysis

Explants from the nasal area (E14), both nasal and basal forebrain areas (E17) and from basal forebrain area (E20) were microdissected from heads of *Gnrh1*-GFP embryos; nose and forebrain cells were dissociated by incubation in 0.05% trypsin with 100 μg/ml DNase I in neurobasal medium (Invitrogen) at 37°C for 15 min. Trypsinization was quenched by addition of neurobasal medium containing 10% heat-inactivated FBS (Invitrogen) at 37°C for 5 min. Cells were washed three times in neurobasal medium (without FBS) to remove serum before FACS and resuspended in neurobasal medium without Phenol Red (Invitrogen) containing L-glutamine (Invitrogen) and B-27 supplement (1:50; Invitrogen). Dissociated cells from eight to ten embryos for each age were pooled for each FACS. FACS was performed by the Wolfson Scientific Support Services (University College London, London, UK) by using a MoFlo Sorter (Dako). A non-green embryo was used as a control for fluorescence. Cells were excited by using a 488-nm Argon laser and detected by using a 530/40 (FL1) bandpass filter. A cell purity of 95-98.5% was obtained for each sort. Sorted (GFP^+^) and unsorted (GFP^−^) cells were directly collected in lysis buffer (Qiagen) and used for RNA extraction.

### Microarray analysis

For GN11 and GT1-7 cells, total RNA was isolated by acid guanidinium thiocyanate-phenol-chloroform extraction (Trizol, Invitrogen) followed by a Qiagen RNeasy kit clean-up procedure (Qiagen). Total RNA from GFP^+^ and GFP^−^ FACS-purified cells was extracted immediately after collection by using a Qiagen RNeasy Plus kit (Qiagen). RNA integrity was verified with an RNA 6000 Nano and Pico kit, and only high-quality RNAs, with RNA integrity number greater than 7 were used. A WT Expression Kit (Invitrogen) and Ovation Pico WTA System V2 (Nugen) were respectively used to prepare sense-strand cDNA. Biotin labeling was performed with a WT Terminal Labeling kit Affymetrix (Invitrogen) and Encore Biotin Module kit (Nugen). Labeled cDNA was hybridized (45°C for 17 h) to the Affymetrix Mouse Gene 1.0 ST and Rat Gene 1.0 ST Gene chips according to the manufacturer's protocol.

### Data processing and visualization

Microarray data analysis, including quality controls, normalization and gene filtering was conducted using AMDA software (Pelizzola et al., 2006). The identification of differentially expressed genes was addressed using linear modeling approach and empirical Bayes methods (Smyth, 2004) together with FDR correction of the *P*-value (Benjamini–Hochberg); the adjusted *P*-value that has been selected in the analysis is <0.05 and log_FC_>2. GO analyses were performed as previously described with reString software (Manzini et al., 2021). Briefly, hierarchical clustering was performed with Euclidean metric on log-transformed ratios of probes fluorescence intensity to average probe intensity (Virtanen et al., 2019 preprint). PCA was performed with Scikit-learn (Pedregosa et al., 2011). Data visualization was performed with SciPy (Virtanen et al., 2019 preprint), matplotlib (Hunter, 2007) and seaborn (Waskom et al., 2022) libraries for the Python programming language.

### Human samples and sequencing

For case 1, exome-sequencing data from 47 patients from a UK GD cohort (CPMS ID 30730) were analyzed. Exome sequencing was performed on DNA extracted from peripheral blood leukocytes, using an Agilent V5 platform and Illumina HiSeq 2000 sequencing. The exome sequences were aligned to the UCSC hg19 reference genome using Burrows-Wheeler Aligner software (BWA-MEM; bwa-0.7.12). Picard tools (picard-tools-1.119) was used to sort alignments and mark PCR duplicates. The genome analysis toolkit (GATK-3.4-46) was used to realign around indels and recalibrate quality scores using dbSNP, Mills and 1000 genomes as reference resources. Variant calling and joint genotyping using pedigree information was performed using HaplotypeCaller in genomic variant call format (GVCF) mode from the genome analysis toolkit. The resulting variants were filtered using the variant quality score recalibration function from GATK. An analysis of the called variants was performed using Ingenuity Variant Analysis (Qiagen). Filtering for potential causal variants was carried out using filters for quality control, allele frequency and predicted functional annotation. Potentially pathogenic variants in candidate genes were verified by Sanger sequencing. Libraries of genomic DNA samples were prepared using a Sureselect Human All Exon v5 kit (Agilent Technologies) and were sequenced on a HiSeq instrument (Illumina) according to the manufacturer's recommendations for paired-end 76-bp reads. The bioinformatics pipeline, alignment processes and quality procedures were as previously described (Yang et al., 2014). Version 3.4-46 of the Genome Analysis Toolkit was used for this study.

For case 2, exome sequencing was realized as previously published (Nambot et al., 2018). Briefly, libraries of genomic DNA samples were prepared using a Human Core Exome kit (Twist Biosciences) and were sequenced on a NovaSeq 6000 instrument (Illumina) according to the manufacturer's recommendations for paired-end 151-bp reads. A mean depth of 86.96× was reached, and 97.2% of the refseq exons were covered at least by ten reads. Variants were identified using a computational platform of the Fédération Hospitalo-Universitaire (FHU) TRANSLAD, hosted by the University of Burgundy Computing Cluster. Raw data quality was evaluated by FastQC software (v0.11.4). Reads were aligned to the GRCh37/hg19 human genome reference sequence using the Burrows-Wheeler Aligner (v0.7.15) (Li and Durbin, 2009). Aligned read data underwent the following steps: (1) duplicate paired-end reads were removed by Picard software (v2.4.1), and (2) base quality score recalibration was done by the Genome Analysis Toolkit (GATK v3.8) Base recalibrator. Using GATK Haplotype Caller, single-nucleotide variants with a quality score >30 and an alignment quality score >20 were annotated with SNPEff (v4.3) (Cingolani et al., 2012). Rare variants were identified by focusing on nonsynonymous changes present at a frequency less than 1% in the GnomAD database. Copy number variants were detected using xHMM (v1.0), annotated using in-house python scripts and then filtered regarding their frequency in public databases (Database of Genomic Variants, International Standards for Cytogenomic Arrays, Deciphering Developmental Disorders).

### Expression vectors and transfection

To introduce the c.163C>T and c.366G>A variants into human *NLGN3* gene, the WT human HA-tagged NLGN3 expression vector (Addgene, 59318) was mutagenized using a QuickChange Lightning Site-Directed Mutagenesis Kit (Agilent Technologies) and specific oligonucleotides for *NLGN3* R55* (fw, 5′-GGCAGTGGTACTCAGGCACCCCTTAGC-3′; rev, 5′-GCTAAGGGGTGCCTGAGTACCACTGCC-3′) and W122* (fw, 5′-GTCATGCTGCCGGTCTGATTCACTGCCAACTTGGATATCG-3′; rev, 5′-TCAGACCGGCAGCATGACTTCGGGCACAGCTGTGTGGATG-3′). To visualize the ER, the mEmerald-ER-3 vector (a gift from Prof. Diego De Stefani; Addgene, 54082) was co-transfected. Expression vectors were transiently transfected (for 24 h) into GN11 cells using Lipofectamine 3000 (Invitrogen).

### Immunoblotting

Cells were lysed in 150 mM NaCl, 50 mM Tris-HCl (pH 7.4) and 1% Triton X-100, supplemented with protease and phosphatase inhibitors (Roche). Then, 20 μg of proteins were transferred to nitrocellulose membranes (Bio-Rad), immunoblotted with rabbit anti-HA (1:1000; Cell Signaling Technology, 3724) and rabbit anti-GAPDH (1:5000; Cell Signaling Technology, 2118), followed by horseradish peroxidase-conjugated anti-rabbit antibody (1:10,000; Santa Cruz Biotechnology, sc-2301).

### Immunocytochemistry

Paraformaldehyde (PFA)-fixed GN11 cells were incubated with PBS containing 10% normal goat serum and 0.1% Triton X-100. Rabbit anti-HA (1:800; Cell Signaling Technology, 3724) and chicken anti-GFP (1:1000; Abcam, ab13970) were used as primary antibodies. Secondary antibodies used were Alexa Fluor 488-conjugated donkey anti-rabbit and anti-chicken and Cy3-conjugated donkey anti-rabbit Fab fragments (1:200; Jackson ImmunoResearch). To detect F-actin, cells were stained with TRITC-conjugated phalloidin (1:400; Sigma-Aldrich, P1951) for 30 min at 37°C (Cannarella et al., 2021). Nuclei were counterstained with DAPI (1:10,000; Sigma-Aldrich, D9542). For immunoperoxidase labeling, cells were incubated with hydrogen peroxide to quench endogenous peroxidase activity before incubation with rabbit anti-NLGN3 (1:200; Budreck and Scheiffele, 2007), followed by biotinylated goat anti-rabbit antibody (1:400; Vector Laboratories, BA-1000), and then developed with an ABC kit (Vector Laboratories, PK6100) and DAB (Sigma-Aldrich, D4293).

### RT-qPCR

Total RNA from GN11, GT1-7 and FACS-sorted cells was retrotranscribed into cDNA as previously described (Busnelli et al., 2020). Briefly, 1 μg of total RNA was reverse transcribed with random hexamers and MultiScribe reverse transcriptase (Applied Biosystems) following the manufacturer's Ginstructions. The expression level of murine *Nlgn3* (fw, 5′-GAAGATGGATCCGGCGCTAA-3′; rev, 5′-ACGATGACGTTGCCGTAACT-3′), *Sema3a* (fw, 5′-CGTCTTCCGGGAACCAACAA-3′; rev, 5′-TGCACAGGCTTTGCCATAGA-3′), *Snai2* (fw, 5′-GAACTGGACACACACACAGTTATT-3′; rev, 5′-TGCCGACGATGTCCATACAG-3′), *L1cam* (fw, 5′-GCTCCTCATCCTGCTCATCC-3′; rev, 5′-TCTCCAGGGACCTGTACTCG-3′) and *Sema3c* (fw, 5′-GAACCCATGTTTGTGGACGC-3′; rev, 5′-CCACCAGTGTCATTAGGGCA-3′) genes was quantified by quantitative PCR (qPCR) on a Bio-Rad CFX Connect thermal cycler with Luna Universal qPCR Master Mix (NEB) in 10 μl reactions, with final concentration of 0.25 μM for each primer. The cycling conditions were 95°C for 1 min, followed by 40 cycles of 15 s at 95°C, 30 s at 60°C and 30 s at 72°C. A final melting curve analysis assured the authenticity of the target product. Triplicate samples were run in all reactions; first-strand DNA synthesis reactions without reverse transcriptase were used as controls. The ΔCq value and the ΔΔCq were calculated relative to control samples using quantification cycle (Cq) threshold values that were normalized to the reference gene, *Gapdh* (fw, 5′-CATCCCAGAGCTGAACG-3′; rev, 5′-CTGGTCCTCAGTGTAGCC-3′).

### Immunostaining

WT C57/Bl6 embryos at E14.5 were fixed for 3 h in 4% PFA and then cryoprotected overnight in 30% sucrose, embedded in OCT and cryosectioned for immunostaining. PFA-fixed tissue sections (25 μm) or cells were incubated with serum-free protein block (Dako, X0909). For immunofluorescence staining, goat anti-NLGN3 (1:25; Santa-Cruz Biotechnology, sc-14091), rabbit anti-GnRH (1:400; Immunostar, 20075), rabbit anti-NLGN3 (1:100; Budreck and Scheiffele, 2007) and goat anti-PLXND1 (1:200; R&D Systems, AF4160) (Cariboni et al., 2015) were used as primary antibodies. Secondary antibodies used were Alexa Fluor 488-conjugated donkey anti-rabbit and Cy3-conjugated donkey anti-goat Fab fragments (1:200; Jackson ImmunoResearch). Nuclei were counterstained with DAPI (1:10,000; Sigma-Aldrich, D9542).

### Image acquisition

Cells were examined with a Zeiss LSM 900 confocal laser scanning microscope equipped with a Zeiss Axiocam 305 color and using a Plan Apochromat 40×1.3 Pil DIC (VIS-IR M27) oil immersion objective (Zeiss). DAPI, Alexa Fluor 488 and Cy3 were excited at 353, 493 and 548 nm and observed at 400-500, 500-540 and 540- 700 nm, respectively. We captured 1024×1024 pixel images in a stepwise fashion over a defined *z*-focus range corresponding to all visible fluorescence within the cell. Maximum projections of the *z*-stack with 0.25 μm optical section were obtained post-acquisition by using Zeiss ZEN System. Adobe Photoshop CS6 software was used to prepare the presented images.

### Quantification

Morphological analyses were performed on confocal images using ImageJ (v1.52a) as previously described (Bouilly et al., 2018) and summarized in Fig. 4D.

### Statistics

Statistical tests employed are outlined in figure legends and were conducted when the experiment had been performed a minimum of three times on a minimum of three individual samples. Data are presented as mean±s.d., and results were considered significant with a *P*-value less than 0.05 (GraphPad Prism 7.0).

### Study approval

Ethical approval for Case 1 was granted by the London-Chelsea National Research Ethics Service committee (13/LO/0257) and the UK NHS Health Research Authority (IRAS 95781). Ethical approval for Case 2 was granted by Ethics Committees of Centre Hospitalier Universitaire de Caen and Centre Hospitalier Universitaire Dijon Bourgogne. All participants provided written informed consent prior to study participation. The study was conducted in accordance with the guidelines of the Declaration of Helsinki. All individual-level data, including clinical data, were de-identified. Participants or their legal representatives gave consent to the publication of the results of this research work in the present study. The animal work was approved by the University of Milan Animal Welfare Body and by the Italian Ministry of Health to A.C. and was conducted in accordance with the EU Directive 2010/63/EU.

## Supplementary Material

10.1242/dmm.049996_sup1 Supplementary information

## References

[DMM049996C1] Abraham, E., Palevitch, O., Gothilf, Y. and Zohar, Y. (2009). The zebrafish as a model system for forebrain GnRH neuronal development. *Gen. Comp. Endocrinol.* 164, 151-160. 10.1016/j.ygcen.2009.01.012 19523393

[DMM049996C2] Al-Jawahiri, R., Foroutan, A., Kerkhof, J., Mcconkey, H., Levy, M., Haghshenas, S., Rooney, K., Turner, J., Shears, D., Holder, M. et al. (2022). SOX11 variants cause a neurodevelopmental disorder with infrequent ocular malformations and hypogonadotropic hypogonadism and with distinct DNA methylation profile. *Genet. Med.* 3600, 1-13. 10.1016/j.gim.2022.02.013 PMC9245088 35341651

[DMM049996C3] Bemben, M. A., Nguyen, T. A., Li, Y., Wang, T., Nicoll, R. A. and Roche, K. W. (2019). Isoform-specific cleavage of neuroligin-3 reduces synapse strength. *Mol. Psychiatry* 24, 145-160. 10.1038/s41380-018-0242-y 30242227

[DMM049996C4] Boehm, U., Bouloux, P.-M., Dattani, M. T., de Roux, N., Dodé, C., Dunkel, L., Dwyer, A. A., Giacobini, P., Hardelin, J.-P., Juul, A. et al. (2015). Expert consensus document: European Consensus Statement on congenital hypogonadotropic hypogonadism--pathogenesis, diagnosis and treatment. *Nat. Rev. Endocrinol.* 11, 547-564. 10.1038/nrendo.2015.112 26194704

[DMM049996C5] Bouilly, J., Messina, A., Papadakis, G., Cassatella, D., Xu, C., Acierno, J. S., Tata, B., Sykiotis, G., Santini, S., Sidis, Y. et al. (2018). DCC/NTN1 complex mutations in patients with congenital hypogonadotropic hypogonadism impair GnRH neuron development. *Hum. Mol. Genet.* 27, 359-372. 10.1093/hmg/ddx408 29202173

[DMM049996C6] Budreck, E. C. and Scheiffele, P. (2007). Neuroligin-3 is a neuronal adhesion protein at GABAergic and glutamatergic synapses. *Eur. J. Neurosci.* 26, 1738-1748. 10.1111/j.1460-9568.2007.05842.x 17897391

[DMM049996C7] Busnelli, M., Manzini, S., Jablaoui, A., Bruneau, A., Kriaa, A., Philippe, C., Arnaboldi, F., Colombo, A., Ferrari, B., Ambrogi, F. et al. (2020). Fat-shaped microbiota affects lipid metabolism, liver steatosis, and intestinal homeostasis in mice fed a low-protein diet. *Mol. Nutr. Food Res.* 64, e1900835. 10.1002/mnfr.201900835 32579743

[DMM049996C8] Cannarella, R., Paganoni, A. J. J., Cicolari, S., Oleari, R., Condorelli, R. A., La Vignera, S., Cariboni, A., Calogero, A. E. and Magni, P. (2021). Anti-müllerian hormone, growth hormone, and insulin-like growth factor 1 modulate the migratory and secretory patterns of GnRH neurons. *Int. J. Mol. Sci.* 22, 2445. 10.3390/ijms22052445 33671044 PMC7957759

[DMM049996C9] Cariboni, A., André, V., Chauvet, S., Cassatella, D., Davidson, K., Caramello, A., Fantin, A., Bouloux, P., Mann, F. and Ruhrberg, C. (2015). Dysfunctional SEMA3E signaling underlies gonadotropin-releasing hormone neuron deficiency in Kallmann syndrome. *J. Clin. Invest.* 125, 2413-2428. 10.1172/JCI78448 25985275 PMC4497752

[DMM049996C10] Cariboni, A., Maggi, R. and Parnavelas, J. G. (2007). From nose to fertility: the long migratory journey of gonadotropin-releasing hormone neurons. *Trends Neurosci.* 30, 638-644. 10.1016/j.tins.2007.09.002 17981344

[DMM049996C11] Casoni, F., Malone, S. A., Belle, M., Luzzati, F., Collier, F., Allet, C., Hrabovszky, E., Rasika, S., Prevot, V., Chédotal, A. et al. (2016). Development of the neurons controlling fertility in humans: new insights from 3D imaging and transparent fetal brains. *Development* 143, 3969-3981. 10.1242/dev.139444 27803058

[DMM049996C12] Chen, J., Bardes, E. E., Aronow, B. J. and Jegga, A. G. (2009). ToppGene Suite for gene list enrichment analysis and candidate gene prioritization. *Nucleic Acids Res.* 37, W305-W311. 10.1093/nar/gkp427 19465376 PMC2703978

[DMM049996C13] Chih, B., Afridi, S. K., Clark, L. and Scheiffele, P. (2004). Disorder-associated mutations lead to functional inactivation of neuroligins. *Hum. Mol. Genet.* 13, 1471-1477. 10.1093/hmg/ddh158 15150161

[DMM049996C14] Cho, H.-J., Shan, Y., Whittington, N. C. and Wray, S. (2019). Nasal placode development, GnRH neuronal migration and Kallmann syndrome. *Front. Cell Dev. Biol.* 7, 1-27. 10.3389/fcell.2019.00121 31355196 PMC6637222

[DMM049996C15] Cingolani, P., Platts, A., Wang, L. L., Coon, M., Nguyen, T., Wang, L., Land, S. J., Lu, X. and Ruden, D. M. (2012). A program for annotating and predicting the effects of single nucleotide polymorphisms. *SnpEff. Fly* 6, 80-92. 10.4161/fly.19695 22728672 PMC3679285

[DMM049996C16] Davey, C., Tallafuss, A. and Washbourne, P. (2010). Differential expression of neuroligin genes in the nervous system of zebrafish. *Dev. Dyn.* 239, 703-714. 10.1002/dvdy.22195 20063411 PMC3071345

[DMM049996C17] Diaczok, D., Divall, S., Matsuo, I., Wondisford, F. E., Wolfe, A. M. and Radovick, S. (2011). Deletion of Otx2 in GnRH neurons results in a mouse model of hypogonadotropic hypogonadism. *Mol. Endocrinol.* 25, 833-846. 10.1210/me.2010-0271 21436260 PMC3082331

[DMM049996C18] Ferreira, L., Silveira, G. and Latronico, A. C. (2013). Approach to the patient with hypogonadotropic hypogonadism. *J. Clin. Endocrinol. Metab.* 98, 1781-1788. 10.1210/jc.2012-3550 23650335

[DMM049996C19] Forni, P. E. and Wray, S. (2015). GnRH, anosmia and hypogonadotropic hypogonadism--where are we? *Front. Neuroendocrinol.* 36, 165-177. 10.1016/j.yfrne.2014.09.004 25306902 PMC4703044

[DMM049996C20] Forni, P. E., Bharti, K., Flannery, E. M., Shimogori, T. and Wray, S. (2013). The indirect role of fibroblast growth factor-8 in defining neurogenic niches of the olfactory/GnRH systems. *J. Neurosci.* 33, 19620-19634. 10.1523/JNEUROSCI.3238-13.2013 24336726 PMC3858631

[DMM049996C21] Gatford, N. J. F., Deans, P. J. M., Duarte, R. R. R., Chennell, G., Sellers, K.J., Raval, P. and Srivastava, D. P. (2022). Neuroligin-3 and neuroligin-4X form nanoscopic clusters and regulate growth cone organization and size. *Hum. Mol. Genet.* 31, 674-691. 10.1093/hmg/ddab277 34542148 PMC8895740

[DMM049996C22] Harrington, J. and Palmert, M. R. (2012). Distinguishing constitutional delay of growth and puberty from isolated hypogonadotropic hypogonadism: critical appraisal of available diagnostic tests. *J. Clin. Endocrinol. Metab.* 97, 3056-3067. 10.1210/jc.2012-1598 22723321

[DMM049996C23] Herbison, A. E. (2016). Control of puberty onset and fertility by gonadotropin-releasing hormone neurons. *Nat. Rev. Endocrinol.* 12, 452-466. 10.1038/nrendo.2016.70 27199290

[DMM049996C24] Hunter, J. D. (2007). Matplotlib: A 2D graphics environment. *Comput. Sci. Eng.* 9, 90-95. 10.1109/MCSE.2007.55

[DMM049996C25] Jamain, S., Quach, H., Betancur, C., Råstam, M., Colineaux, C., Gillberg, I. C., Soderstrom, H., Giros, B., Leboyer, M., Gillberg, C. et al. (2003). Mutations of the X-linked genes encoding neuroligins NLGN3 and NLGN4 are associated with autism. *Nat. Genet.* 34, 27-29. 10.1038/ng1136 12669065 PMC1925054

[DMM049996C26] Kato, M., Ui-Tei, K., Watanabe, M. and Sakuma, Y. (2003). Characterization of voltage-gated calcium currents in gonadotropin-releasing hormone neurons tagged with green fluorescent protein in rats. *Endocrinology* 144, 5118-5125. 10.1210/en.2003-0213 12960038

[DMM049996C27] Keen, K.,L., Petersen, A. J., Figueroa, A. G., Fordyce, B. I., Shin, J., Yadav, R., Erdin, S., Pearce, R. A., Talkowski, M. E., Bhattacharyya, A. et al. (2021). Physiological characterization and transcriptomic properties of GnRH neurons derived from human stem cells. *Endocrinology* 162, 1-26. 10.1210/endocr/bqab120 PMC8294693 34125902

[DMM049996C28] Li, H. and Durbin, R. (2009). Fast and accurate short read alignment with Burrows-Wheeler transform. *Bioinformatics* 25, 1754-1760. 10.1093/bioinformatics/btp324 19451168 PMC2705234

[DMM049996C29] Lund, C., Yellapragada, V., Vuoristo, S., Balboa, D., Trova, S., Allet, C., Eskici, N., Pulli, K., Giacobini, P., Tuuri, T. et al. (2020). Characterization of the human GnRH neuron developmental transcriptome using a GNRH1-TdTomato reporter line in human pluripotent stem cells. *Dis. Model. Mech.* 13, dmm040105. 10.1242/dmm.040105 31996360 PMC7075073

[DMM049996C30] Maggi, R., Pimpinelli, F., Molteni, L., Milani, M., Martini, L. and Piva, F. (2000). Immortalized luteinizing hormone-releasing hormone neurons show a different migratory activity in vitro. *Endocrinology* 141, 2105-2112. 10.1210/endo.141.6.7494 10830297

[DMM049996C31] Maione, L., Dwyer, A. A., Francou, B., Guiochon-Mantel, A., Binart, N., Bouligand, J. and Young, J. (2018). GENETICS IN ENDOCRINOLOGY: Genetic counseling for congenital hypogonadotropic hypogonadism and Kallmann syndrome: new challenges in the era of oligogenism and next-generation sequencing. *Eur. J. Endocrinol.* 178, R55-R80. 10.1530/EJE-17-0749 29330225

[DMM049996C32] Manfredi-Lozano, M., Leysen, V., Adamo, M., Paiva, I., Rovera, R., Pignat, J.-M., Timzoura, F. E., Candlish, M., Eddarkaoui, S., Malone, S. A. et al. (2022). GnRH replacement rescues cognition in Down syndrome. *Science* 377, eabq4515. 10.1126/science.abq4515 36048943 PMC7613827

[DMM049996C33] Manzini, S., Busnelli, M., Colombo, A., Franchi, E., Grossano, P. and Chiesa, G. (2021). reString: an open-source Python software to perform automatic functional enrichment retrieval, results aggregation and data visualization. *Sci. Rep.* 11, 23458. 10.1038/s41598-021-02528-0 34873191 PMC8648753

[DMM049996C34] Messina, A., Langlet, F., Chachlaki, K., Roa, J., Rasika, S., Jouy, N., Gallet, S., Gaytan, F., Parkash, J., Tena-Sempere, M. et al. (2016). A microRNA switch regulates the rise in hypothalamic GnRH production before puberty. *Nat. Neurosci.* 19, 835-844. 10.1038/nn.4298 27135215

[DMM049996C35] Nambot, S., Thevenon, J., Kuentz, P., Duffourd, Y., Tisserant, E., Bruel, A.-L., Mosca-Boidron, A.-L., Masurel-Paulet, A., Lehalle, D., Jean-Marçais, N. et al. (2018). Clinical whole-exome sequencing for the diagnosis of rare disorders with congenital anomalies and/or intellectual disability: substantial interest of prospective annual reanalysis. *Genet. Med.* 20, 645-654. 10.1038/gim.2017.162 29095811

[DMM049996C36] Nguyen, T. A., Lehr, A. W. and Roche, K. W. (2020). Neuroligins and Neurodevelopmental Disorders: X-Linked Genetics. *Front. Synaptic Neurosci.* 12, 33. 10.3389/fnsyn.2020.00033 32848696 PMC7431521

[DMM049996C37] Ohlsson Gotby, V., Söder, O., Frisén, L., Serlachius, E., Bölte, S., Almqvist, C., Larsson, H., Lichtenstein, P. and Tammimies, K. (2019). Hypogonadotrophic hypogonadism, delayed puberty and risk for neurodevelopmental disorders. *J. Neuroendocrinol.* 31, e12803. 10.1111/jne.12803 31630461

[DMM049996C38] Oleari, R., Caramello, A., Campinoti, S., Lettieri, A., Ioannou, E., Paganoni, A., Fantin, A., Cariboni, A. and Ruhrberg, C. (2019). PLXNA1 and PLXNA3 cooperate to pattern the nasal axons that guide gonadotropin-releasing hormone neurons. *Development* 146, dev176461. 10.1242/dev.176461 31690636

[DMM049996C39] Oleari, R., Massa, V., Cariboni, A. and Lettieri, A. (2021). The differential roles for neurodevelopmental and neuroendocrine genes in shaping GnRH neuron physiology and deficiency. *Int. J. Mol. Sci.* 22, 9425. 10.3390/ijms22179425 34502334 PMC8431607

[DMM049996C40] Paraoanu, L. E., Becker-Roeck, M., Christ, E. and Layer, P. G. (2006). Expression patterns of neurexin-1 and neuroligins in brain and retina of the chick embryo: Neuroligin-3 is absent in retina. *Neurosci. Lett.* 395, 114-117. 10.1016/j.neulet.2005.10.076 16300891

[DMM049996C41] Parkash, J., Messina, A., Langlet, F., Cimino, I., Loyens, A., Mazur, D., Gallet, S., Balland, E., Malone, S. A., Pralong, F. et al. (2015). Semaphorin7A regulates neuroglial plasticity in the adult hypothalamic median eminence. *Nat. Commun.* 6, 6385. 10.1038/ncomms7385 25721933 PMC4351556

[DMM049996C42] Pedregosa, F., Varoquaux, G., Gramfort, A., Michel, V., Thirion, B., Grisel, O., Blondel, M., Prettenhofer, P., Weiss, R., Dubourg, V. et al. (2011). Scikit-learn: machine learning in {P}ython. *J. Mach. Learn. Res* 12, 2825-2830.

[DMM049996C43] Pelizzola, M., Pavelka, N., Foti, M. and Ricciardi-Castagnoli, P. (2006). AMDA: An R package for the automated microarray data analysis. *BMC Bioinformatics* 7, 1-9. 10.1186/1471-2105-7-335 16824223 PMC1534071

[DMM049996C44] Quartier, A., Courraud, J., Thi Ha, T., Mcgillivray, G., Isidor, B., Rose, K., Drouot, N., Savidan, M.-A., Feger, C., Jagline, H. et al. (2019). Novel mutations in NLGN3 causing autism spectrum disorder and cognitive impairment. *Hum. Mutat.* 40, 2021-2032. 10.1002/humu.23836 31184401

[DMM049996C45] Redin, C., Gérard, B., Lauer, J., Herenger, Y., Muller, J., Quartier, A., Masurel-Paulet, A., Willems, M., Lesca, G., El-Chehadeh, S. et al. (2014). Efficient strategy for the molecular diagnosis of intellectual disability using targeted high-throughput sequencing. *J. Med. Genet.* 51, 724-736. 10.1136/jmedgenet-2014-102554 25167861 PMC4215287

[DMM049996C46] Richards, S., Aziz, N., Bale, S., Bick, D., Das, S., Gastier-Foster, J., Grody, W. W., Hegde, M., Lyon, E., Spector, E. et al. (2015). Standards and guidelines for the interpretation of sequence variants: a joint consensus recommendation of the American College of Medical Genetics and Genomics and the Association for Molecular Pathology. *Genet. Med.* 17, 405-423. 10.1038/gim.2015.30 25741868 PMC4544753

[DMM049996C47] Saengkaew, T., Patel, H. R., Banerjee, K., Butler, G., Dattani, M. T., Mcguigan, M., Storr, H. L., Willemsen, R. H., Dunkel, L. and Howard, S. R. (2021). Genetic evaluation supports differential diagnosis in adolescent patients with delayed puberty. *Eur. J. Endocrinol.* 185, 617-627. 10.1530/EJE-21-0387 34403359 PMC8558847

[DMM049996C48] Smyth, G. K. (2004). Linear models and empirical bayes methods for assessing differential expression in microarray experiments. *Stat. Appl. Genet. Mol. Biol.* 3, 1-25. 10.2202/1544-6115.1027 16646809

[DMM049996C49] Sobreira, N., Schiettecatte, F., Valle, D. and Hamosh, A. (2015). GeneMatcher: a matching tool for connecting investigators with an interest in the same gene. *Hum. Mutat.* 36, 928-930. 10.1002/humu.22844 26220891 PMC4833888

[DMM049996C50] Swee, D. S. and Quinton, R. (2019). Congenital hypogonadotrophic hypogonadism: Minipuberty and the case for neonatal diagnosis. *Front. Endocrinol.* 10, 97. 10.3389/fendo.2019.00097 PMC6393341 30846970

[DMM049996C51] Uchigashima, M., Cheung, A. and Futai, K. (2021). Neuroligin-3: a circuit-specific synapse organizer that shapes normal function and autism spectrum disorder-associated dysfunction. *Front. Mol. Neurosci.* 14, 1-22. 10.3389/fnmol.2021.749164 PMC8526735 34690695

[DMM049996C52] Varghese, M., Keshav, N., Jacot-Descombes, S., Warda, T., Wicinski, B., Dickstein, D. L., Harony-Nicolas, H., De Rubeis, S., Drapeau, E., Buxbaum, J. D. et al. (2017). Autism spectrum disorder: neuropathology and animal models. *Acta Neuropathol.* 134, 537-566. 10.1007/s00401-017-1736-4 28584888 PMC5693718

[DMM049996C53] Varoqueaux, F., Aramuni, G., Rawson, R. L., Mohrmann, R., Missler, M., Gottmann, K., Zhang, W., Südhof, T. C. and Brose, N. (2006). Neuroligins determine synapse maturation and function. *Neuron* 51, 741-754. 10.1016/j.neuron.2006.09.003 16982420

[DMM049996C54] Virtanen, P., Gommers, R., Oliphant, T.E., Haberland, M., Reddy, T., Cournapeau, D., Burovski, E., Peterson, P., Weckesser, W., Bright, J. et al. (2019). SciPy 1.0--fundamental algorithms for scientific computing in python. *arXiv* arXiv:1907.10121. 10.48550/arXiv.1907.10121 PMC7056644 32015543

[DMM049996C55] Wang, Y., Madhusudan, S., Cotellessa, L., Kvist, J., Eskici, N., Yellapragada, V., Pulli, K., Lund, C., Vaaralahti, K., Tuuri, T. et al. (2022). Deciphering the transcriptional landscape of human pluripotent stem cell-derived GnRH neurons: the role of Wnt signaling in patterning the neural fate. *Stem Cells* 54, 1-54. 10.1093/stmcls/sxac069 PMC9806769 36153707

[DMM049996C56] Waskom, M., Botvinnik, O., O'Kane, D., Hobson, P., Ostblom, J., Lukauskas, S., Gemperline, D., Tom, A., Halchenko, Y., Cole, J. et al. (2022). seaborn. 10.5281/zenodo.592845

[DMM049996C57] Xu, J., Du, Y.-L., Xu, J.-W., Hu, X.-G., Gu, L.-F., Li, X.-M., Hu, P.-H., Liao, T.-L., Xia, Q.-Q., Sun, Q. et al. (2019). Neuroligin 3 regulates dendritic outgrowth by modulating Akt/mTOR signaling. *Front. Cell. Neurosci.* 13, 518. 10.3389/fncel.2019.00518 31849609 PMC6896717

[DMM049996C58] Yang, Y., Muzny, D. M., Xia, F., Niu, Z., Person, R., Ding, Y., Ward, P., Braxton, A., Wang, M., Buhay, C. et al. (2014). Molecular findings among patients referred for clinical whole-exome sequencing. *JAMA* 312, 1870. 10.1001/jama.2014.14601 25326635 PMC4326249

