# Supplementary material for: Autism-linked NLGN3 is a key regulator of gonadotropin-releasing hormone deficiency
Source: Dis Model Mech. 2023 Mar 28;16(3):dmm049996. doi: 10.1242/dmm.049996 (PMC10110398; doi:10.1242/dmm.049996)
Supplement: Supplementary information [file dmm-16-049996-s1.pdf]

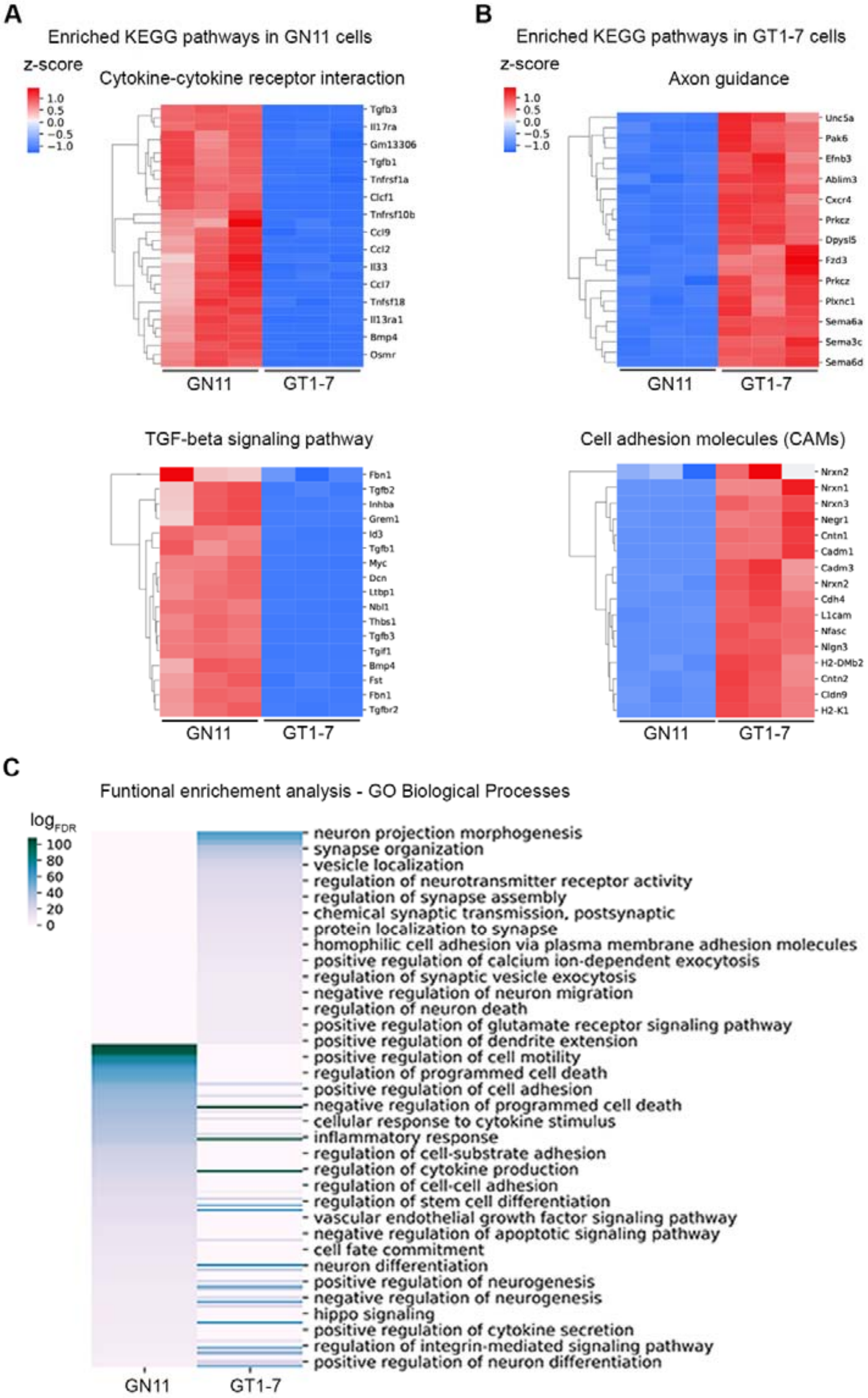

Disease Models & Mechanisms • Supplementary information

Fig. S1. GN11 and GT1-7 cells displayed specific gene expression signatures. A,B – Z-scored gene expression values for genes belonging to selected KEGG pathways are shown. Heat-maps representing examples of color-coded expression levels of genes belonging to significantly enriched KEGG pathways at GN11 (A) and GT1-7 (B) cells. C – Enriched GO Biological Processes (FDR < 10^{-5}) found by STRING functional enrichment analysis computed on DEGs between GN11 and GT1-7 cells. Enrichment scores are reported as logFDR; higher values (deep blue) indicate highly enriched pathways, lower values (grey) indicate poorly enriched pathways.

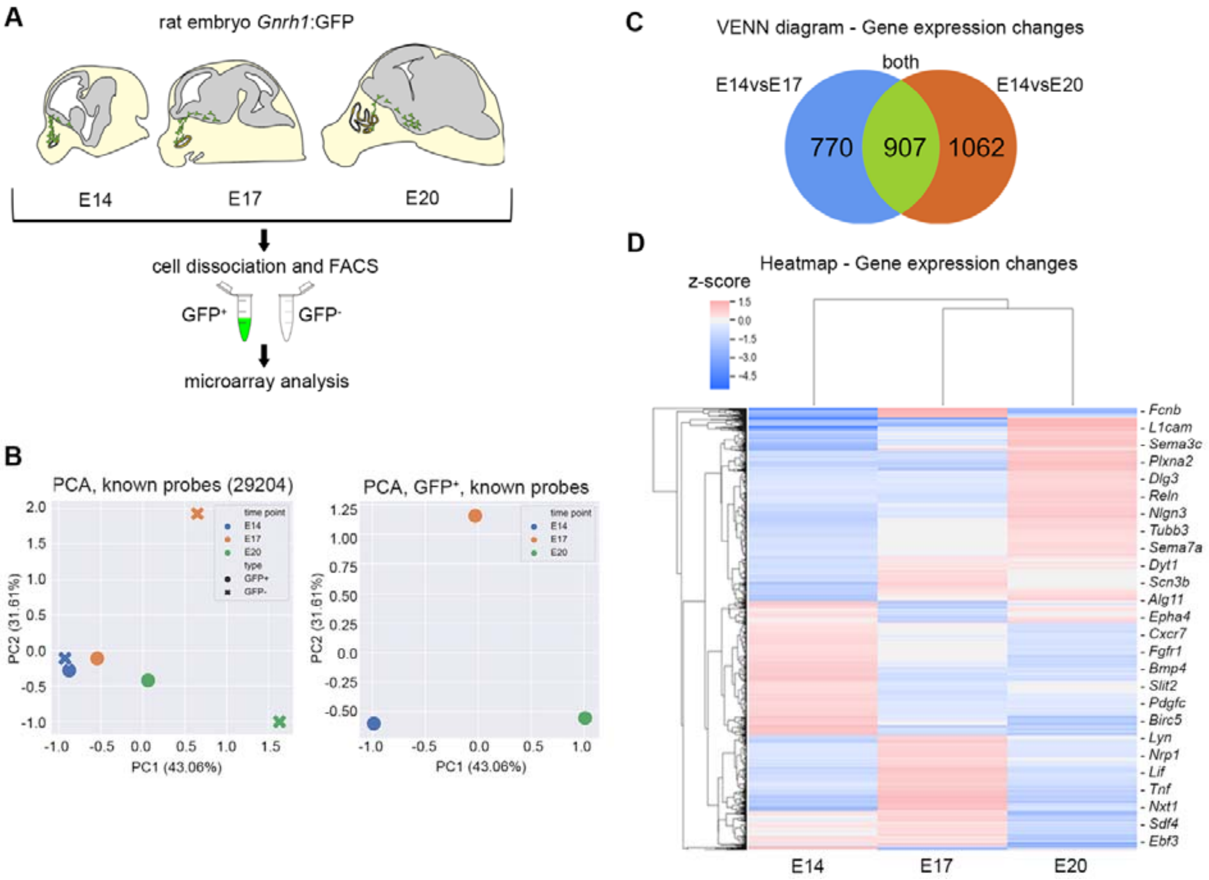

Fig. S2. Transcriptomic analysis of GnRH neurons from *Gnrh1*-GFP rats at different developmental time points. A - Schematic drawing representing *Gnrh1*-GFP rat embryos at indicated developmental time points and localization of GnRH neurons (in green). GFP^+ cells were isolated after embryo dissociation with FACS and RNA from GFP^+ cells were used for microarray analysis. B - Dimensionality reduction was performed on gene expression space for each sample, and the first 2 principal coordinates are charted. Samples are shown for each time point (E14 - blue, E17 - orange, E20 - green). Circles represent GFP^+ cells, crosses GFP^- cells. PCA investigation showed that the developmental stage strongly impacted on gene expression signatures, with samples clustering away one from each other. C - The intersection of genes showing expression differences in excess of 2 fold change in the comparison E14 vs E17 (blue) and E14 vs E20 (orange) is shown as a Venn diagram. In green, genes that exhibited expression changes in both E17 and E20 vs the common E14 baseline. D - The ratio of the log2 expression value and mean value across all samples for each probe has been calculated and clustered hierarchically (Euclidean distance metric). Red indicates higher expression, blue lower expression. The heatmap reveals three broad gene clusters.

Disease Models & Mechanisms • Supplementary information

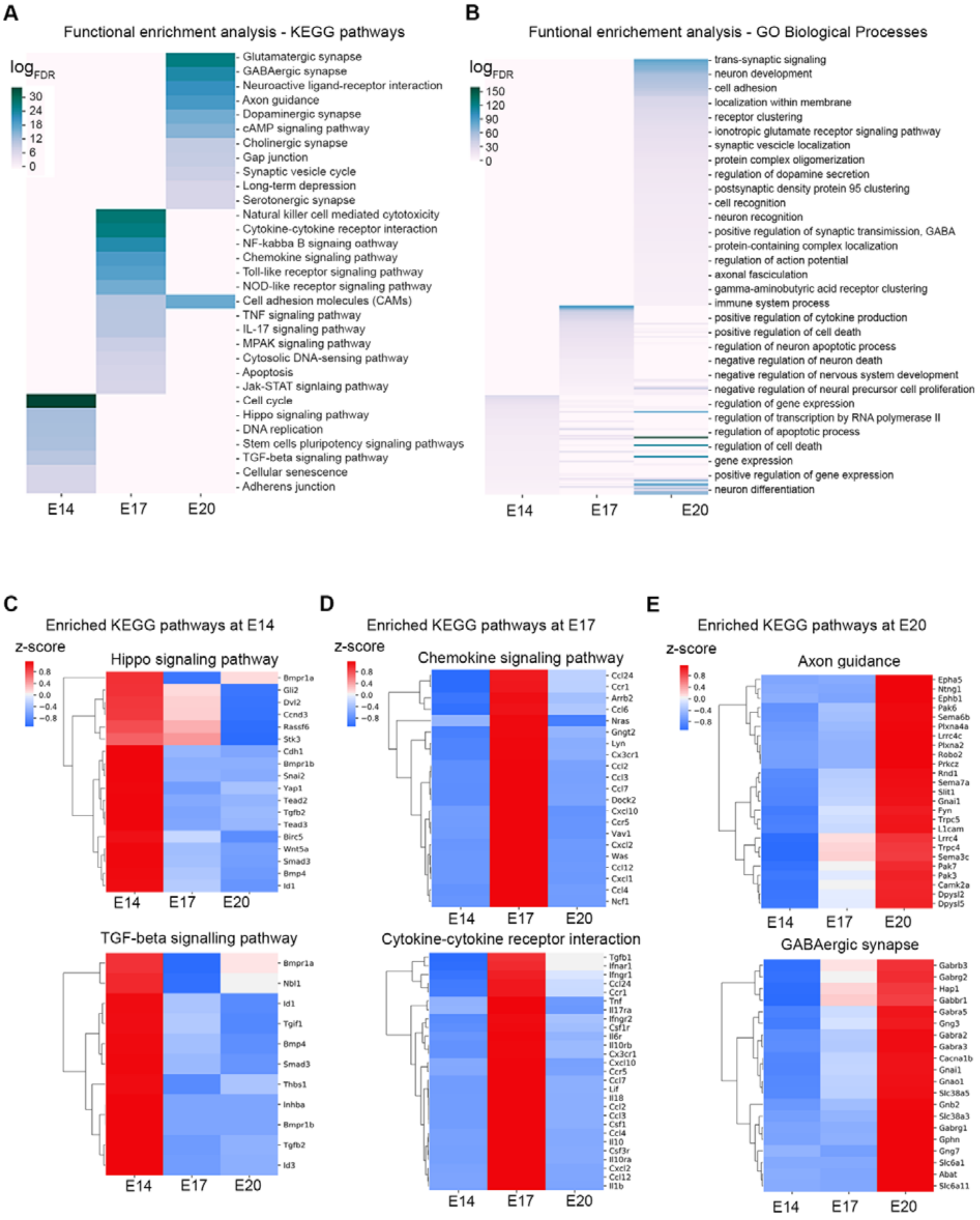
A
Functional enrichment analysis - KEGG pathways
log FDR
30
24
18
12
6
0
Glutamatergic synapse
GABAergic synapse
Neuroactive ligand-receptor interaction
Axon guidance
Dopaminergic synapse
cAMP signaling pathway
Cholinergic synapse
Gap junction
Synaptic vesicle cycle
Long-term depression
Serotonergic synapse
Natural killer cell mediated cytotoxicity
Cytokine-cytokine receptor interaction
NF-kabba B signaling oathway
Chemokine signaling pathway
Toll-like receptor signaling pathway
NOD-like receptor signaling pathway
Cell adhesion molecules (CAMs)
TNF signaling pathway
IL-17 signaling pathway
MPAK signaling pathway
Cytosolic DNA-sensing pathway
Apoptosis
Jak-STAT signlaing pathway
Cell cycle
Hippo signaling pathway
DNA replication
Stem cells pluripotency signaling pathways
TGF-beta signaling pathway
Cellular senescence
Adherens junction
E14
E17
E20
B
Funtional enrichement analysis - GO Biological Processes
log FDR
150
120
90
60
30
0
trans-synaptic signaling
neuron development
cell adhesion
localization within membrane
receptor clustering
ionotropic glutamate receptor signaling pathway
synaptic vescicle localization
protein complex oligomerization
regulation of dopamine secretion
postsynaptic density protein 95 clustering
cell recognition
neuron recognition
positive regulation of synaptic tranismission, GABA
protein-containing complex localization
regulation of action potential
axonal fasciculation
gamma-aminobutyric acid receptor clustering
immune system process
positive regulation of cytokine production
positive regulation of cell death
regulation of neuron apoptotic process
negative regulation of neuron death
negative regulation of nervous system development
negative regulation of neural precursor cell proliferation
regulation of gene expression
regulation of transcription by RNA polymerase II
regulation of apoptotic process
regulation of cell death
gene expression
positive regulation of gene expression
neuron differentiation
E14
E17
E20
C
Enriched KEGG pathways at E14
z-score
Hippo signaling pathway
TGF-beta signalling pathway
E14
E17
E20
D
Enriched KEGG pathways at E17
z-score
Chemokine signaling pathway
Cytokine-cytokine receptor interaction
E14
E17
E20
E
Enriched KEGG pathways at E20
z-score
Axon guidance
GABAergic synapse
E14
E17
E20

Fig. S3. GFP$^+$ cells displayed specific gene expression signatures for each developmental stage. A,B - Functional enrichment analysis has been carried out for genes upregulated at each time point. Enriched (FDR < 0.01) KEGG pathways (A) and GO Biological Processes (B) are summarized in an heatmap. Enrichment scores are reported as $\log_{FDR}$; higher values (deep blue) indicate highly enriched pathways, lower values (grey) indicate poorly enriched pathways. C-E - Z-scored gene expression values for genes belonging to selected KEGG pathways are shown. Heat-maps representing examples of color-coded expression levels of genes belonging to selected enriched KEGG pathways at E14 (C), E17 (D) and E20 (E).

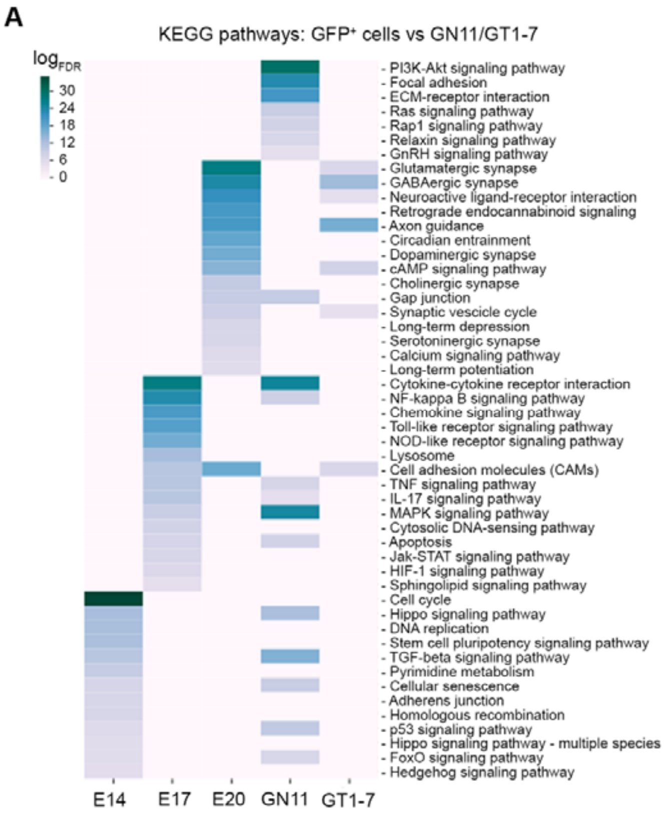

Fig. S4. Functional enrichment analyses revealed overlapping enriched pathways at early and late stages of development, respectively. A - The functional enrichment analysis detailed in Figure 1E is integrated with genes from primary GFP^+ cells at E14, E17 and E20. Genes upregulated in GN11 cells vs GT1-7 are enriched in the same pathways (FDR < 0.01) as GFP^+ cells at E14 and E17. Notably, a subset of enriched pathways specific to GN11 cells exists. Conversely, genes upregulated in GT1-7 cells mostly enriched in the same pathways as GFP^+ cells at E20. Enrichment scores are reported as $\log_{FDR}$; higher values (deep blue) indicate highly enriched pathways, lower values (grey) indicate poorly enriched pathways.

Disease Models & Mechanisms • Supplementary information

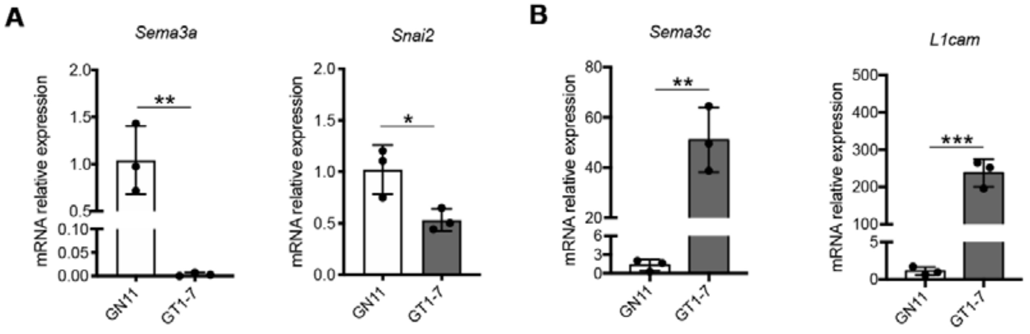

Fig. S5. Validation of candidate genes by RT-qPCR. A,B – Expression levels of 2 representative early (A) and late (B) genes from the top 20 candidate gene lists (Table 3) quantified by qPCR. *Sema3a* and *Snai2* were significantly upregulated in GN11 cells, whereas *Sema3c* and *L1cam* were significantly upregulated in GT1-7 cells. Data are presented as mean ± SD of 3 biological replicates. P values indicate Student's t test (* P < 0.05, ** P < 0.01, *** P < 0.001).

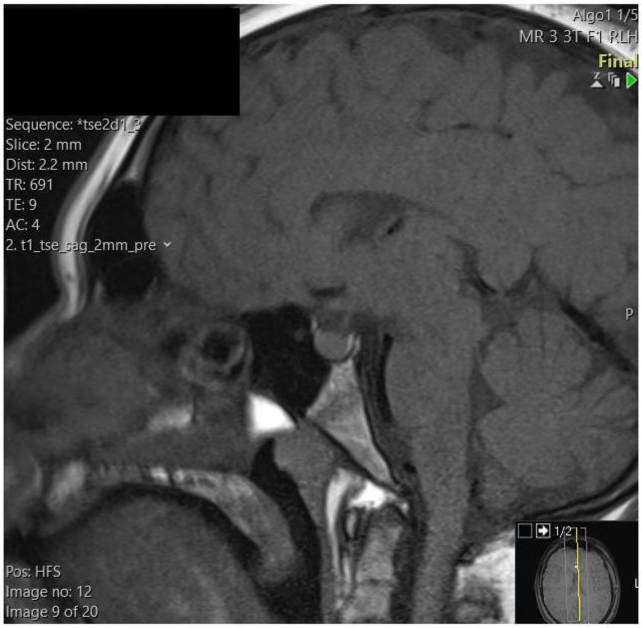

Fig. S6. MRI scan of Case 1 pituitary gland. MRI pituitary (sagittal image) showing a normally sized and located pituitary gland with posterior bright spot, and anatomically normal surrounding tissues.

Table S1. List of selected KEGG pathways (FDR < 0.01)

Term ID	Term description	Observed gene count	Background gene count	FDR
**GN11 cells**				
mmu04510	Focal adhesion	26	196	4.99E-07
mmu04060	Cytokine-cytokine receptor interaction	29	279	4.91E-06
mmu04512	ECM-receptor interaction	16	87	4.91E-06
**GT1-7 cells**				
mmu04360	Axon guidance	24	176	6.02E-05
mmu04514	Cell adhesion molecules	15	155	0.0438

Disease Models & Mechanisms • Supplementary information

Table S2. List of selected GO Biological Processes (FDR < 10^{-5})

Term ID	Term description	Observed gene count	Background gene count	FDR
**GN11 cells**				
GO:0030334	Regulation of cell migration	117	915	1.08E-32
GO:2000145	Regulation of cell motility	120	963	1.16E-32
GO:0040012	Regulation of locomotion	122	1010	4.07E-32
GO:2000147	Positive regulation of cell motility	81	589	6.13E-24
GO:0030335	Positive regulation of cell migration	79	567	1.21E-23
GO:0030155	Regulation of cell adhesion	78	705	6.14E-18
GO:0040011	Locomotion	104	1183	1.38E-17
GO:0016477	Cell migration	85	845	2.46E-17
GO:0048870	Cell motility	89	971	6.65E-16
GO:0007155	Cell adhesion	74	744	9.13E-15
GO:0045785	Positive regulation of cell adhesion	51	425	8.83E-13
GO:0034097	Response to cytokine	71	818	1.58E-11
GO:0050920	Regulation of chemotaxis	32	226	1.43E-09
GO:0006935	Chemotaxis	47	487	6.72E-09
GO:0030029	Actin filament-based process	48	551	8.52E-08
GO:0030036	Actin cytoskeleton organization	45	496	8.73E-08
GO:0007162	Negative regulation of cell adhesion	32	283	1.68E-07
GO:0050921	Positive regulation of chemotaxis	23	150	1.71E-07
GO:0010810	Regulation of cell-substrate adhesion	27	213	3.06E-07
GO:0030336	Negative regulation of cell migration	29	267	1.73E-06
GO:0040013	Negative regulation of locomotion	31	314	4.04E-06
GO:0019221	Cytokine-mediated signaling pathway	29	295	1.04E-05
GO:0060326	Cell chemotaxis	23	197	1.15E-05
GO:0032970	Regulation of actin filament-based process	34	388	1.22E-05
GO:0022407	Regulation of cell-cell adhesion	35	412	1.58E-05
GO:0010811	Positive regulation of cell-substrate adhesion	18	132	2.68E-05
GO:0031589	Cell-substrate adhesion	20	171	5.60E-05
GO:0007015	Actin filament organization	24	243	8.11E-05
**GT1-7 cells**				
GO:0031175	Neuron projection development	94	697	2.97E-22
GO:0048812	Neuron projection morphogenesis	77	479	5.59E-22
GO:0030030	Cell projection organization	117	1124	7.86E-20
GO:0061564	Axon development	63	386	2.64E-18
GO:0007409	Axonogenesis	60	351	3.04E-18

GO:0010975	Regulation of neuron projection development	79	607	5.86E-18
GO:0007411	Axon guidance	43	234	6.73E-14
GO:0040011	Locomotion	96	1183	3.11E-10
GO:0031346	Positive regulation of cell projection organization	52	474	3.63E-09
GO:0006935	Chemotaxis	52	487	8.63E-09
GO:0010976	Positive regulation of neuron projection development	43	366	2.50E-08
GO:0050770	Regulation of axonogenesis	31	203	3.14E-08
GO:0007155	Cell adhesion	65	744	7.01E-08
GO:0098609	Cell-cell adhesion	43	398	2.15E-07
GO:0010977	Negative regulation of neuron projection development	27	177	3.80E-07
GO:0048870	Cell motility	70	971	1.45E-05
GO:0016477	Cell migration	62	845	3.99E-05
GO:0007156	Homophilic cell adhesion via plasma membrane adhesion molecules	17	99	4.79E-05
GO:0050771	Negative regulation of axonogenesis	14	67	5.99E-05

Table S3. List of known HH/KS causative genes used as 'input' genes to instruct ToppGene software

HH/KS genes	
ANOS1	*NR0B1*
AMH	*NRP1*
AMHR2	*NRP2*
AXL	*NSMF*
CCDC141	*NTN1*
CHD7	*OTUD4*
CHL1	*PCSK1*
DCC	*PLXNA1*
DMXL2	*PLXNA3*
DUSP6	*PNPLA6*
FEZF1	*POLR3A*
FGF17	*POLR3B*
FGF8	*PROK2*
FGFR1	*PROKR2*
FLRT3	*RNF216*
FSHB	*SEMA3A*
GNRH1	*SEMA3E*
GNRHR	*SEMA3F*
GLI3	*SEMA7A*
HS6ST1	*SMCHD1*
IGSF10	*SOX10*
IL17RD	*SOX2*
KISS1	*SPRY4*
KISS1R	*STUB1*
KLB	*TAC3*
LEP	*TACR3*
LEPR	*TUBB3*
LHB	*WDR11*
NDNF	*TCF12*

Table S4. List of variants with CADD > 25 and MAF < 1% found in the GD cohort

Gene	Chr	Position	nt sub	aa sub	SIFT	Polyphen	CADD	MAF %
NLGN3	X	70367965	c.366G>A	p.W122*	NA	NA	36	NA
CLSTN2	3	140167490	c.917T>A	p.I306N	Damaging	Possibly damaging	26.4	0.353
PLXNC1	12	94543619	c.872G>C	p.R291P	Damaging	Probably damaging	31	NA

Disease Models & Mechanisms • Supplementary information
